# Oxygen-Atom Defect Formation in Polyoxovanadate Clusters via Proton-Coupled Electron Transfer

**DOI:** 10.1021/jacs.1c13432

**Published:** 2022-03-11

**Authors:** Eric Schreiber, Alex A. Fertig, William W. Brennessel, Ellen M. Matson

**Affiliations:** Department of Chemistry, University of Rochester, Rochester, New York 14627, United States

## Abstract

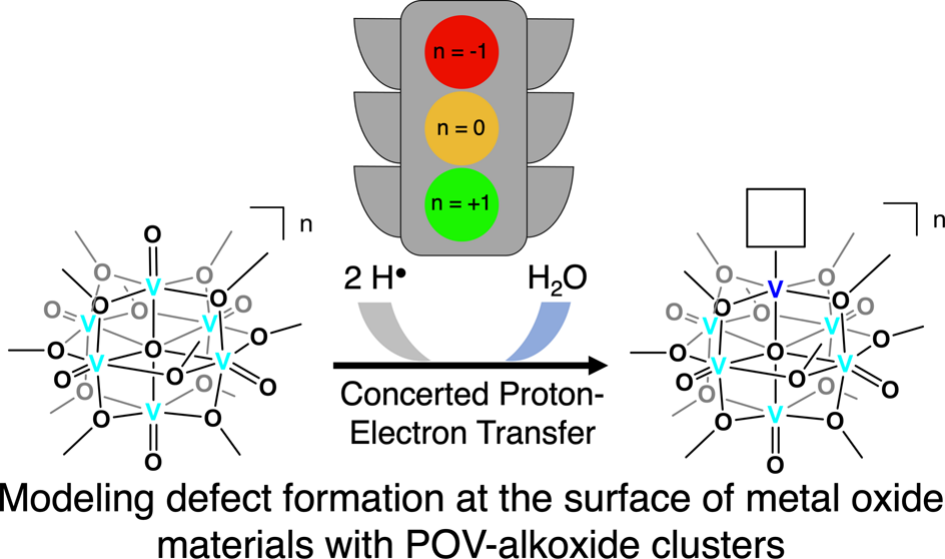

The uptake of hydrogen atoms (H-atoms) into reducible metal oxides has implications in catalysis and energy storage. However, outside of computational modeling, it is difficult to obtain insight into the physicochemical factors that govern H-atom uptake at the atomic level. Here, we describe oxygen-atom vacancy formation in a series of hexavanadate assemblies via proton-coupled electron transfer, presenting a novel pathway for the formation of defect sites at the surface of redox-active metal oxides. Kinetic investigations reveal that H-atom transfer to the metal oxide surface occurs through concerted proton–electron transfer, resulting in the formation of a transient V^III^–OH_2_ moiety that, upon displacement of the water ligand with an acetonitrile molecule, forms the oxygen-deficient polyoxovanadate-alkoxide cluster. Oxidation state distribution of the cluster core dictates the affinity of surface oxido ligands for H-atoms, mirroring the behavior of reducible metal oxide nanocrystals. Ultimately, atomistic insights from this work provide new design criteria for predictive proton-coupled electron-transfer reactivity of terminal M=O moieties at the surface of nanoscopic metal oxides.

## Introduction

Hydrogen atom (H-atom) uptake in reducible metal oxides has emerged as a popular route for doping materials with implications in catalysis, energy storage, and energy conversion. H-atom insertion has been accomplished through a number of methods, including hydrogen spillover^1−3^ and the codoping of electrons and protons.^4−6^ More recently, the concerted transfer of protons and electrons (i.e., proton-coupled electron transfer or PCET) from molecular donors has been identified as an alternative means for the incorporation of proton/electron pairs into metal oxides,^7−9^ which enables the activation of inert substrates and materials by bypassing energetically costly intermediates.^10,11^ Thus, the development of systems, which involve PCET to and/or from these materials, presents as an exciting area of research, with implications in the development of novel reactivity at metal oxide surfaces.

**Figure 1:**
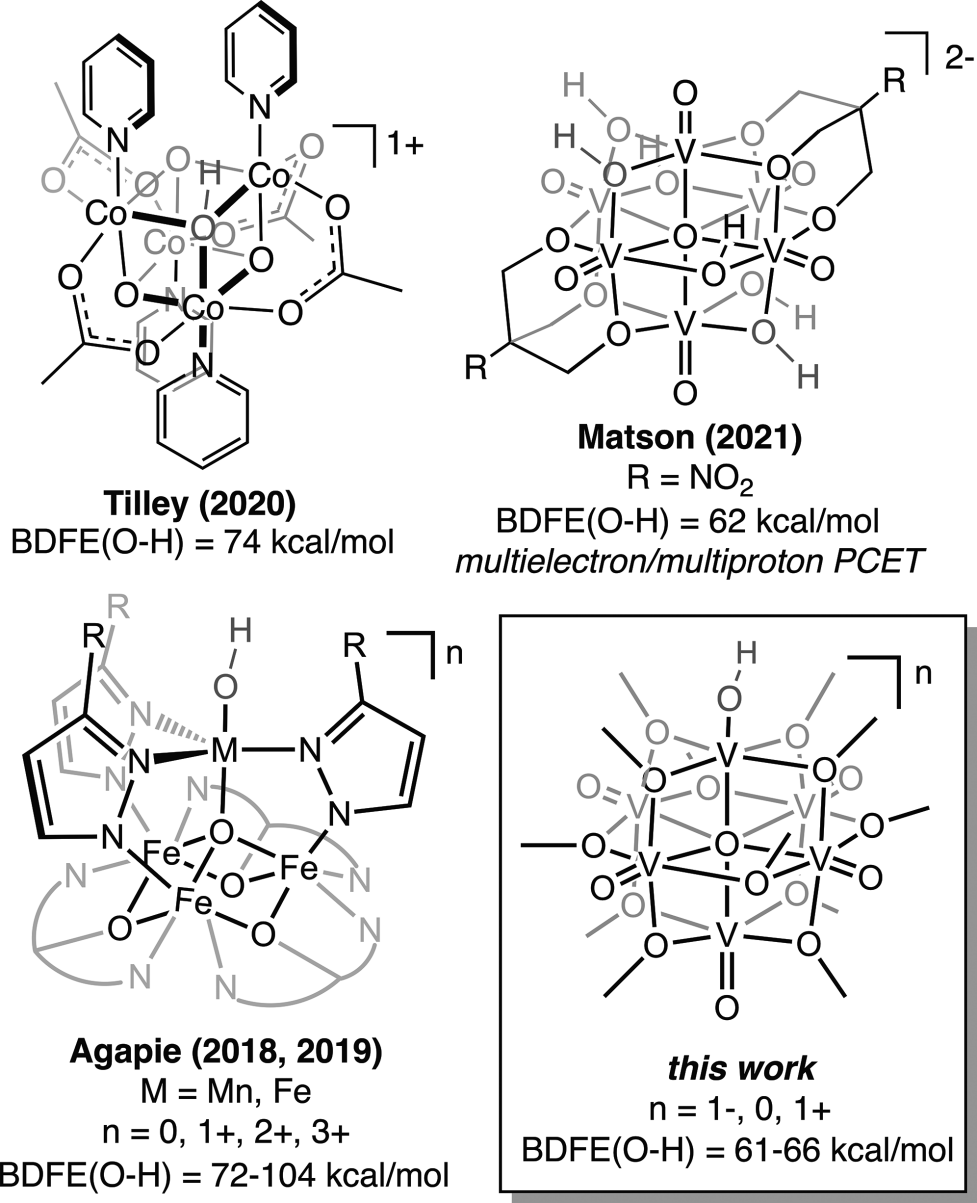
Select examples of proton-coupled electron-transfer reactivity of molecular metal oxide clusters.

While the dynamics of proton and electron transfer at metal oxide surfaces have been studied extensively in the field of electrochemical energy storage,^12,13^ researchers have only very recently begun to understand how these processes play out on molecular length scales.^14,15^ Seminal work from Tilley has described H-atom uptake in cobalt oxide cubanes, demonstrating that these assemblies function as potent oxidants capable of facilitating the C–H bond activation (Figure 1).^16^ Additionally, iron oxide dimers have been shown to direct H-atom reactivity toward the oxido moiety spanning the two metal centers.^17^ More recently, reports from our group^18^ (Figure 1) and others^19^ describe the ability of multimetallic vanadium oxide complexes to facilitate the activation of E–H (E = O, N, C) bonds via PCET. Notably, all of these examples have focused on PCET to bridging oxide moieties, resulting in the formation of surface μ_2_-OH ligands.

By comparison, a few investigations have probed the reactivity of *terminal* oxido moieties in multinuclear molecular MO*_x_* assemblies. This gap in knowledge is striking, as the thermochemistry of H-atom transfer (HAT) to these surface sites is critical for elucidating the mechanisms of oxygen-atom (O-atom) defect formation in reducible metal oxides. In a series of articles targeting the understanding of HAT of relevance to C–H oxidation in enzymes, Agapie and co-workers reported the activation of M=O moieties in multinuclear molecular clusters supported by a multidentate ligand (Figure 1).^20,21^ In these works, the progression of the apical metal ion (M = Fe, Mn) from a metal-aquo species to a high-valent terminal metal oxido, via an isolable metal-hydroxide intermediate, is probed. The authors note that the oxidation-state distribution of the distal metal ions has a dramatic impact on the HAT reactivity of the site-differentiated metal center. More recently, Tilley and co-workers have shown that a terminal Ru^V^-oxo embedded within a cobalt oxide cubane is able to facilitate the C–H oxidation of organic substrates via H-atom abstraction. However, due to the instability of the proposed Ru^III^–OH_2_ product, the authors were unable to determine the precise thermochemical parameters of HAT in this system.^22^

In an effort to understand how surface-based reactivity at metal oxides translates across the periodic table to early transition metal complexes, our research group is studying proton and H-atom uptake in polyoxovanadate-alkoxides (POV-alkoxides). Upon introduction of protons to the dianionic assembly, [V_6_^IV^O_7_(OR)_12_]^2–^, the formation of an O-atom vacancy at the surface of the polyoxovanadate is observed.^23,24^ These studies provide a distinct perspective on mechanisms of acid-induced surface activation of nanoscopic metal oxide materials, as these clusters lack the nucleophilic surface bridging oxido moieties commonly invoked in charge compensation following the reduction of POMs (i.e., protonation). This attribute of POV-alkoxides allows for control over site-specificity in PCET by directing reactivity to terminal oxido moieties.

**Scheme 1:**
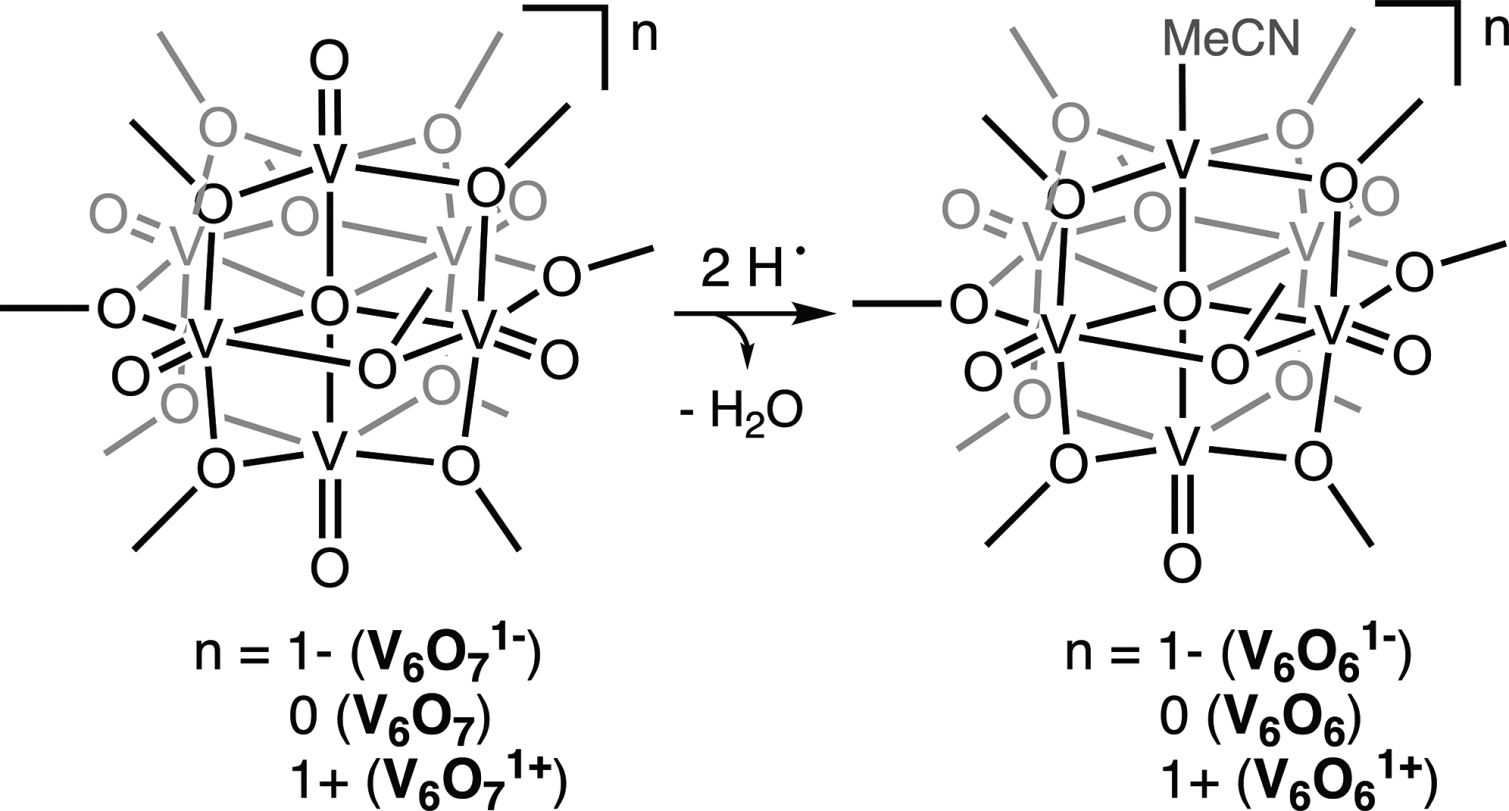
H-Atom Transfer to POV-Alkoxide Clusters Studied in This Work Scheme includes key, associating presented structures with labels used throughout the work.

Herein, we describe PCET to a series of POV-alkoxides, [V_6_O_7_(OCH_3_)_12_]*^n^* (*n* = 1–, 0, 1+); reduction of a single, terminal vanadyl moiety occurs upon the addition of 2 equiv of H-atoms, resulting in the formation of an O-atom vacancy (Scheme 1). Our studies reveal that more oxidized POV-alkoxides are able to abstract H-atom equivalents from substrates with stronger E–H bonds, indicating that the oxidation state distribution of distal vanadium centers influences the reactivity of a single site in the Lindqvist ion. We additionally summarize a series of kinetic investigations that give insight into the mechanism of HAT in these systems; activation of a terminal V=O bond proceeds through a rate-limiting concerted proton–electron-transfer step. Eyring analysis reveals that the entropy of activation is sensitive to the p*K*_a_ of substrate and basicity of the cluster surface, indicating preorganization of H-atom donor and acceptor prior to HAT. This work provides atomistic insight into the role terminal oxido moieties play in the activation of metal oxide surfaces by HAT.

## Results and Discussion

### Reactivity of H-Atom Donors with **V**_**6**_**O**_**7**_^**1–**^

The reactivity of systems in which electrons and protons are transferred as pairs has been shown to rely on the relative strengths of the E–H bonds (where E = O, N, C, etc.) that are broken and formed during the course of the reaction.^25^ As such, quantification of the E–H bond free energy helps predict the reactivity of systems. A common approach for the determination of E–H bond strengths relies on methods popularized by Bordwell.^26^ Using thermochemical parameters involved in the individual steps of proton and electron transfer, the strength of the E–H bond can be determined using eq 1, in terms of the bond dissociation free energy (BDFE)
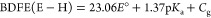
1where *E*° is the standard reduction potential, p*K*_a_ is the acid dissociation constant, and *C*_g_ is a constant that relates to the reduction potential of H^+^/H^•^ in the solvent of interest (*C*_g_ = 52.6 kcal/mol in acetonitrile, MeCN^27^).

**Figure 2:**
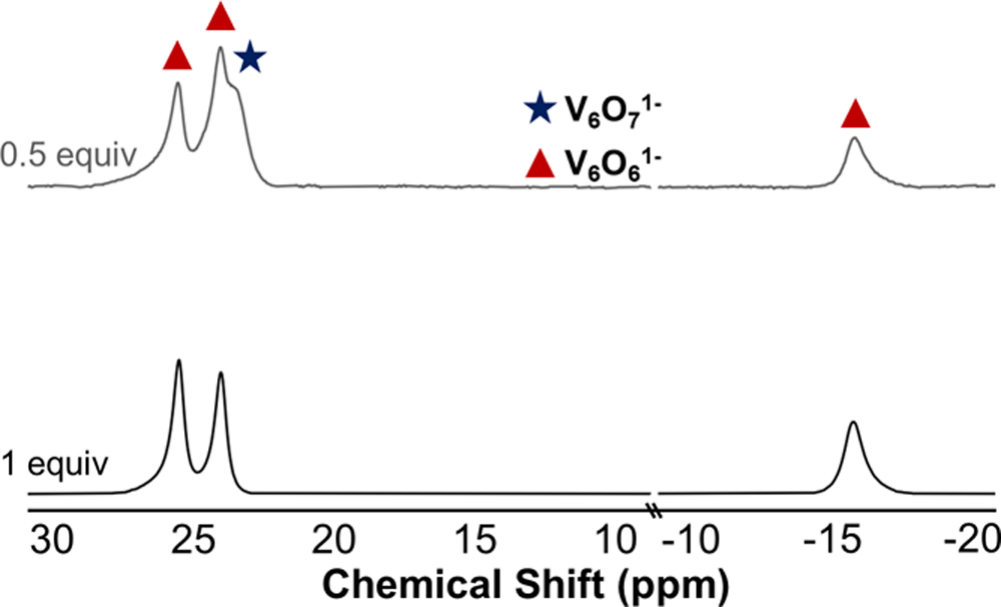
^1^H NMR spectra of reactions between **V**_**6**_**O**_**7**_^**1–**^ and various equivalents of H_2_Phen (0.5 equiv, top; 1 equiv, bottom) in CD_3_CN at 21 °C.

Previous work has reported that the surface basicity of POV-alkoxides is dependent on the oxidation state of the assembly, two factors that influence the BDFE(E–H), as outlined by the Bordwell equation.^24^ This observation led us to hypothesize that terminal oxido moieties in vanadium oxide clusters might also be reactive with H-atoms. To directly probe the reactivity of POV-alkoxides with H-atom donors, we first investigated the reactivity of [V_6_O_7_(OCH_3_)_12_]^1–^ (**V**_**6**_**O**_**7**_^**1–**^) with 5,10-dihydrophenazine (H_2_Phen; BDFE(N–H) avg. = 58.7 kcal/mol;^27^Scheme 1). This substrate has the weakest E–H bonds of those studied in this work and thus seemed an appropriate starting point. While, in principle, HAT would result in the formation of [V_6_O_6_(OH)(OCH_3_)_12_]^1–^, the established instability of the hydroxide species translates to an expected product distribution of an equimolar mixture of the fully oxygenated parent cluster (**V**_**6**_**O**_**7**_^**1–**^) and the O-atom vacant product [V_6_O_6_(OCH_3_)_12_(MeCN)]^1–^ (**V**_**6**_**O**_**6**_^**1–**^).^23,24^ Upon the addition of H_2_Phen to **V**_**6**_**O**_**7**_^**1–**^, a rapid color change from green to brown was observed. Analysis of the product by proton nuclear magnetic resonance (^1^H NMR) spectroscopy revealed four paramagnetically shifted and broadened resonances (Figure 2): one signal is located at 23.4 ppm, corresponding to **V**_**6**_**O**_**7**_^**1–**^, with the remaining three resonances found at 25.3, 23.9, and −15.6 ppm consistent with the formation of **V**_**6**_**O**_**6**_^**1–**^.^28^

While consistent with our proposed mechanism of hydroxide-substituted POV-alkoxide disproportionation,^23,24^ the observed reactivity with one H-atom equivalent leaves half an equivalent of starting material in the reaction mixture. Thus, we hypothesized that the addition of a full equivalent H_2_Phen to **V**_**6**_**O**_**7**_^**1–**^, correlating to the availability of two H-atom equivalents, might result in the complete conversion of the fully oxygenated POV-alkoxide cluster to its oxygen-deficient congener. Exposure of 1 equiv of H_2_Phen to a solution of **V**_**6**_**O**_**7**_^**1–**^ in MeCN results in a rapid color change from dark green to deep pink. The reaction reaches completion within 45 min, with the sole product identified as **V**_**6**_**O**_**6**_^**1–**^ (96% yield; Figure 2, bottom). The anticipated byproducts of this reaction, phenazine and water, are also observed in the ^1^H NMR spectrum of the crude reaction mixture (Figure S1).

**Figure 3:**
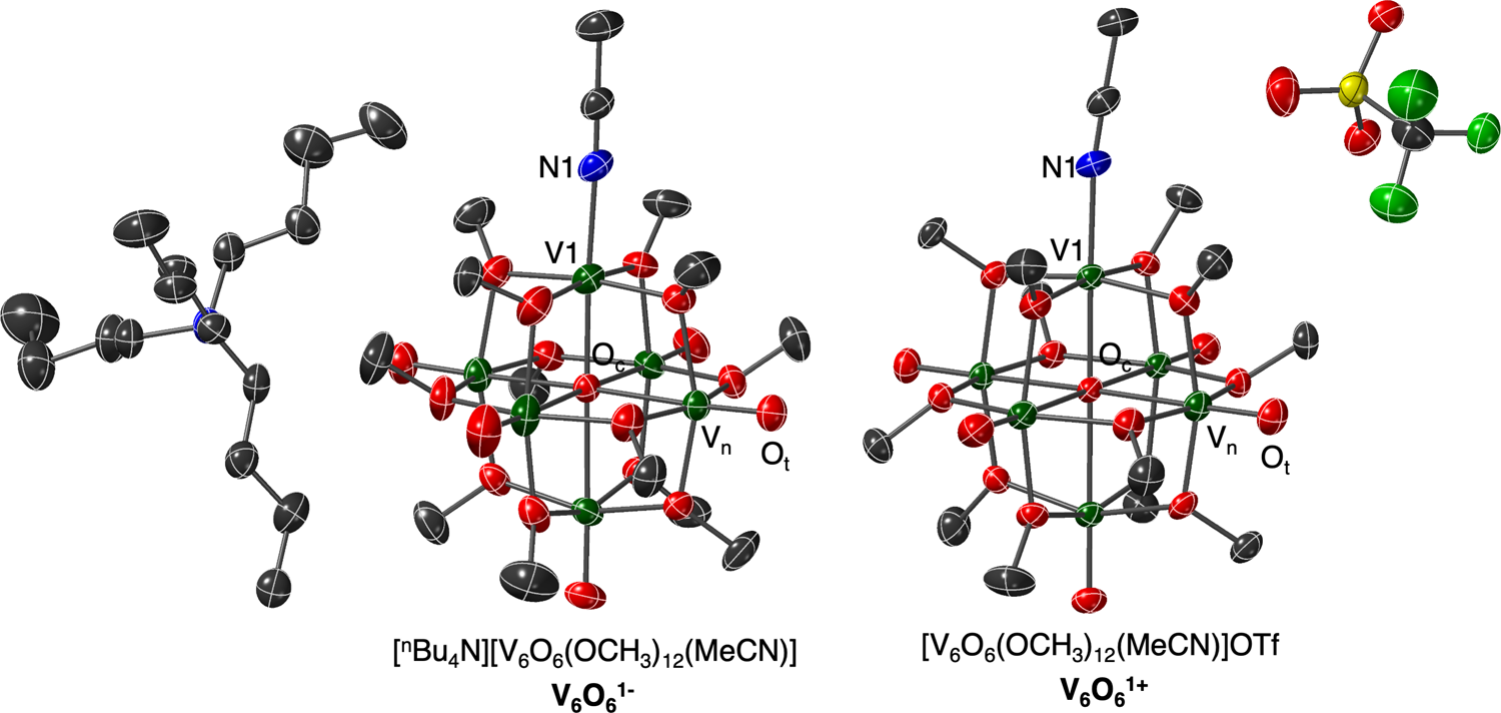
Molecular structures of **V**_**6**_**O**_**6**_^**1–**^ and **V**_**6**_**O**_**6**_^**1+**^ shown as 50% probability ellipsoids. Key: V, green; N, blue; O, red; and C, gray. H-atoms are removed for clarity.

The molecular structure of **V**_**6**_**O**_**6**_^**1–**^ has not been previously reported, despite significant efforts by our research group. However, the improved yield of the oxygen-deficient cluster accessed via HAT provided a sample with sufficient purity for the growth of single crystals suitable for X-ray analysis (Figure 3 and Tables 1 and S1; see the Supporting Information for additional information). Refinement of the data revealed the expected speciation for **V**_**6**_**O**_**6**_^**1–**^, where the cluster features a single, O-atom-deficient vanadium center, with the defect site saturated by a coordinated MeCN molecule. The site-differentiated vanadium center exhibits a short V1–O_c_ bond (2.068(4) Å; O_c_ = μ_6_-oxido moiety located in the center of the Lindqvist ion); this observation is consistent with trends noted in V1–O_c_ bond lengths in previously reported oxygen-deficient POV-alkoxide clusters.^28−30^ Notably, as each vanadium is located on a general position within the unit cell, the assignment of individual oxidation states of metal centers can be accomplished through bond valence sum (BVS) calculations (Table S2). These calculations corroborate the experimentally determined oxidation-state distribution of **V**_**6**_**O**_**6**_^**1–**^ (V^III^V_5_^IV^).^28^

**Table 1:** Selected Bond Lengths and Angles for **V**_**6**_**O**_**7**_^**1–**^,^47^**V**_**6**_**O**_**6**_^**1–**^, **V**_**6**_**O**_**7**_^**1+**^,^47^ and **V**_**6**_**O**_**6**_^**1+**^ Found from Their Solid-State Molecular Structures

bond	V_6_O_7_^1–^*n* = 1–3	V_6_O_6_^1–^*n* = 2–6	V_6_O_7_^1+^*n* = 1–4	V_6_O_6_^1+^*n* = 2–6
V1–N1		2.115(7) Å		2.089(4) Å
V1–O_c_^a^		2.068(4) Å		2.083(3) Å
V_n_–O_t_ (avg)^b^,^c^	1.606 Å	1.600 Å	1.577 Å	1.590 Å
V_n_–O_c_ (avg)^a^,^b^	2.311 Å	2.333 Å	2.274 Å	2.313 Å

a O_c_ = μ_6_ central O-atom.

b V_n_ = vanadyl ions within the Lindqvist core.

c O_t_ = terminal oxido ligands.

The oxidation-state distribution observed in **V**_**6**_**O**_**6**_^**1–**^ presents an intriguing analogy to studies on proton–electron co-doping in vanadium dioxide. The net incorporation of H-atoms into the extended VO_2_ lattice has been shown to generate V^III^ centers throughout the material, as observed in X-ray photoelectron spectroscopy (XPS) and XANES analyses.^4,5^ This indicates the reduction of both the metal centers and corresponding M–O bonds upon the addition of hydrogen equivalents. The authors hypothesize that the H-atom uptake results in the formation of V–OH moieties within the lattice of the doped material, supported by infrared analyses that reveal the formation of O–H bonds following electron–proton codoping of VO_2_. Our work with POV-alkoxides similarly reveals the formation of a V^III^ center following HAT to the cluster surface. However, through the use of single-crystal X-ray diffraction, the atomic precision in our analysis of the products of H-atom uptake in POV-alkoxide clusters indicates the formation of an oxygen vacancy following reduction. This result suggests that the product speciation of H-atom-doped VO_2_ could alternatively include metal-aquo species at the O-atom deficient sites.

**Figure 4:**
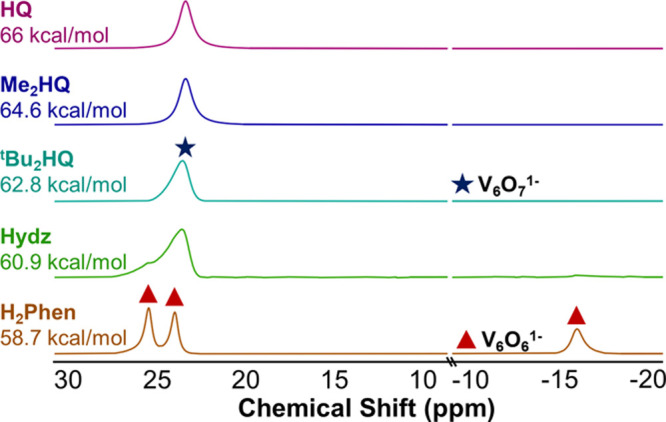
^1^H NMR spectra of reactions between **V**_**6**_**O**_**7**_^**1–**^ and HAT reagents in CD_3_CN at 21 °C.

Next, we investigated the HAT reactivity of **V**_**6**_**O**_**7**_^**1–**^ with additional substrates possessing stronger E–H bonds (e.g., 2,6-di-*tert*-butyl-1,4-hydroquinone, ^t^Bu_2_HQ, BDFE(OH): 62.8 kcal/mol; 2,6-dimethyl-1,4-hydroquinone, Me_2_HQ, BDFE(OH): 64.6 kcal/mol; 1,4-hydroquinone, HQ, BDFE(OH): 67.3 kcal/mol)^27^ in an attempt to benchmark the thermodynamics of HAT to the monoanionic POV-alkoxide cluster. Upon the exposure of **V**_**6**_**O**_**7**_^**1–**^ to the aforementioned substrates, no reaction was observed. In the case of the reaction of **V**_**6**_**O**_**7**_^**1–**^ with hydrazobenzene (Hydz; average BDFE(N–H) = 60.9 kcal mol^–1^),^27^ the addition of an equivalent of substrate results in the formation of a small amount of the vacancy product (Figure 4). We hypothesized that the poor conversion at room temperature might be due to the comparatively high BDFE(N–H) describing the first HAT event from Hydz to the cluster, resulting in a prohibitively large activation barrier for defect formation under ambient conditions. Indeed, running the reaction at elevated temperatures reveals the quantitative formation of **V**_**6**_**O**_**6**_^**1–**^ after 72 h (Figure S2).

**Scheme 2:**
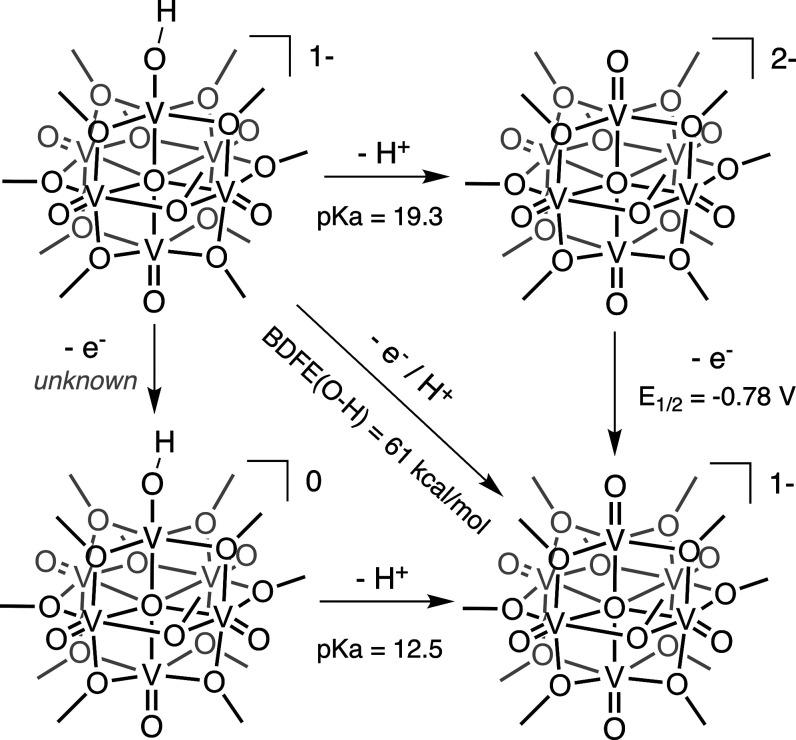
Theoretical Thermochemical Square Scheme for a 1e-/1H+ Transfer to **V**_**6**_**O**_**7**_^**1–**^ Using the reduction potential of **V**_**6**_**O**_**7**_^**1–**^ and an approximated p*K*_a_ of [V_6_O_6_(OH)(OCH_3_)_12_]^1–^, we estimate the strength of the surface V(O–H) moiety of [V_6_O_6_(OH)(OCH_3_)_12_]^1–^ to be 61 kcal/mol.

The various extents of HAT reactivity between the monoanionic cluster and organic substrates possessing a range of BDFE(E–H) values suggest that thermodynamics dictates this type of reactivity at the terminal vanadyl sites. As such, we predict that the strength of the transiently formed hydroxide species possesses a BDFE(O–H) in the range of 60–62 kcal/mol based on the reactivity observed in Figure 2. In an attempt to better quantify this value, we turned to methods popularized by Bordwell via eq 1, where reduction potentials and acid dissociation constants required to form the hydroxide species (Scheme 2) allow for precise determination of the bond strength formed at the vanadyl site. While the reduction potential of POV-alkoxide clusters has been reported by our group and others, acid dissociation constants for the purported hydroxide-substituted assembly are unable to be found due to their instability. Indeed, describing proton uptake in terms of equilibrium constants is not possible due to the rapid disproportionation of the acidified cluster. To work around this constraint, we turn to previous work from our group that has shown the direct relationship between the oxidation state of the cluster and its affinity for protons in acetonitrile.^24^ In this work, the reactivity of the fully oxygenated cluster, [V_6_O_7_(OCH_3_)_12_]^2–^, with organic acids of varying strengths (p*K*_a_ = 22.6–12.5) was evaluated; the addition of acids of p*K*_a_ values higher than 18 revealed incomplete conversion of [V_6_O_7_(OCH_3_)_12_]^2–^ to the disproportionation products, **V**_**6**_**O**_**7**_^**1–**^ and **V**_**6**_**O**_**6**_^**1–**^. Under the assumption that the conversion of half of [V_6_O_7_(OCH_3_)_12_]^2–^ corresponds to a “half-way” point for cluster acidification, we can approximate the p*K*_a_ of the hydroxide-substituted species, [V_6_O_6_(OH)(OCH_3_)_12_]^1–^. Using the reduction potential of **V**_**6**_**O**_**7**_^**1–**^ (−0.78 V) and the approximated p*K*_a_ of [V_6_O_6_(OH)(OCH_3_)_12_]^1–^ (19.3), we estimate the BDFE(O–H) of [V_6_O_6_(OH)(OCH_3_)_12_]^1–^ to be 61 kcal/mol (Scheme 2 and Table 2). The predicted BDFE(O–H) for [V_6_O_6_(OH)(OCH_3_)_12_]^1–^ is broadly consistent with its observed reactivity with organic H-atom donors.

**Table 2:** Thermochemical Parameters Used to Find Theoretical Bond Dissociation Free Energies for Transient Hydroxide-Substituted POV-Alkoxide Clusters^a^

compound^a^	p*K*_a_^24^	*E*_1/2_ (vs Fc^+/0^)^27^	BDFE(O–H)
[V_6_O_6_(OH)(OCH_3_)_12_]^1–^	19.3	–0.78 V	61 kcal/mol
[V_6_O_6_(OH)(OCH_3_)_12_]^0^	12.5	–0.28 V	63 kcal/mol
[V_6_O_6_(OH)(OCH_3_)_12_]^1+^	5.5	0.25 V	66 kcal/mol

a Refer to Scheme 2 for the relevant square scheme of the PCET reaction.

**Figure 5:**
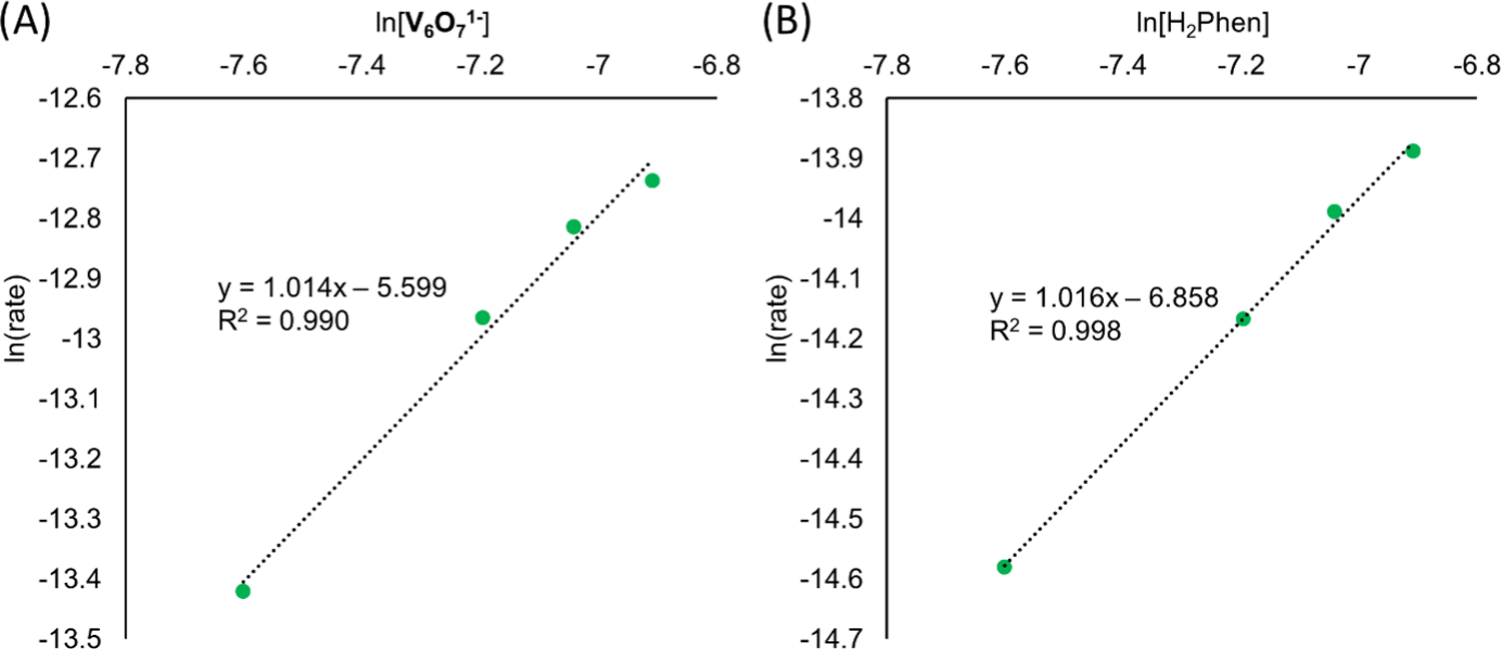
Natural log of the rate plotted against the natural log of the concentration of each reactant in the reduction of **V**_**6**_**O**_**7**_^**1–**^ by H_2_Phen. All reactions are performed in MeCN at −5 °C. (A) The changes in the rate of formation of **V**_**6**_**O**_**6**_^**1–**^ as the concentration of **V**_**6**_**O**_**7**_^**1–**^ is varied from 0.5 to 1 mM. The concentration of H_2_Phen is held at 5 mM. The slope of ∼1 indicates an order of 1 for the reaction. (B) The changes in the rate of formation of the cluster **V**_**6**_**O**_**6**_^**1–**^ as the concentration of H_2_Phen is varied from 0.5 to 1 mM. The concentration of **V**_**6**_**O**_**7**_^**1–**^ is held at 5 mM. The slope of ∼1 indicates an order of 1 for the reaction.

While the determination of thermochemical values, such as BDFE(E–H), allows for the driving force of a reaction to be predicted, this information is not sufficient for the elucidation of the HAT reaction pathway. Given the fact that mechanistic variations of PCET in metal oxides can result in significant consequences in the kinetics of surface-mediated processes,^25^ we performed further analysis to determine the mechanism by which the oxygen-deficient product is generated. Both the parent cluster, **V**_**6**_**O**_**7**_^**1–**^, and the product, **V**_**6**_**O**_**6**_^**1–**^, are distinguishable via ^1^H NMR spectroscopy. As such, we are able to establish the rate of the reaction by monitoring changes in the concentration of the product as a function of time (Figure 5; see the Experimental Section for additional details). Initial experiments focused on establishing a rate expression for defect formation, allowing for insight into the rate-limiting step of the reaction between the monoanionic cluster and the HAT reagent, H_2_Phen. Using pseudo-first order reaction conditions, the order with respect to each reactant can be determined. In terms of both the cluster and HAT reagent, the slopes of the plot of the natural log of rate vs the natural log of concentration indicate an order of 1, resulting in the rate expression seen in eq 2
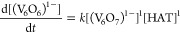
2The experimentally determined rate expression indicates that the rate-limiting step of the O-atom vacancy formation via HAT involves the reaction of 1 equiv of the monoanionic cluster and 1 equiv of the reductant. However, an understanding of the specific sequence of electron and proton transfer from the reductant to the cluster requires additional experimentation. In general, PCET occurs in one of three simple mechanisms:^25,31^ (1) the proton can be transferred in an initial step, followed by the electron (typically referred to as proton transfer–electron transfer, or PT–ET), (2) the electron can be transferred first followed by the proton in electron transfer–proton transfer (ET–PT), and (3) both electron and proton are transferred in a single kinetic step, known as concerted proton–electron transfer (CPET). Understanding which pathway PCET occurs is vital for developing efficient HAT systems.

A common tool used to narrow the possible pathways by which PCET occurs is kinetic isotope effect (KIE) experiments. These studies are designed to probe the involvement of hydrogen in the rate-limiting step; upon isotopic substitution, a significant decrease in the rate of reaction is observed if hydrogen is transferred during the rate-limiting step of a given reaction.^32^ When repeating rate analysis using D_2_Phen a substantial decrease in the rate of formation of **V**_**6**_**O**_**6**_^**1–**^ is observed (*k*_obs-D_ = 5 × 10^–4^). The KIE value of 2.1 (*k*_obs-H_/*k*_obs-D_) eliminates the possibility for an ET–PT pathway (Figure S3). We can additionally eliminate the PT–ET pathway, as the mild basicity of the cluster surface prohibits direct protonation of the assembly by H_2_Phen.^24^ This leaves the CPET pathway as the most likely mechanism for the transfer of a H-atom equivalent to **V**_**6**_**O**_**7**_^**1–**^.

**Scheme 3:**
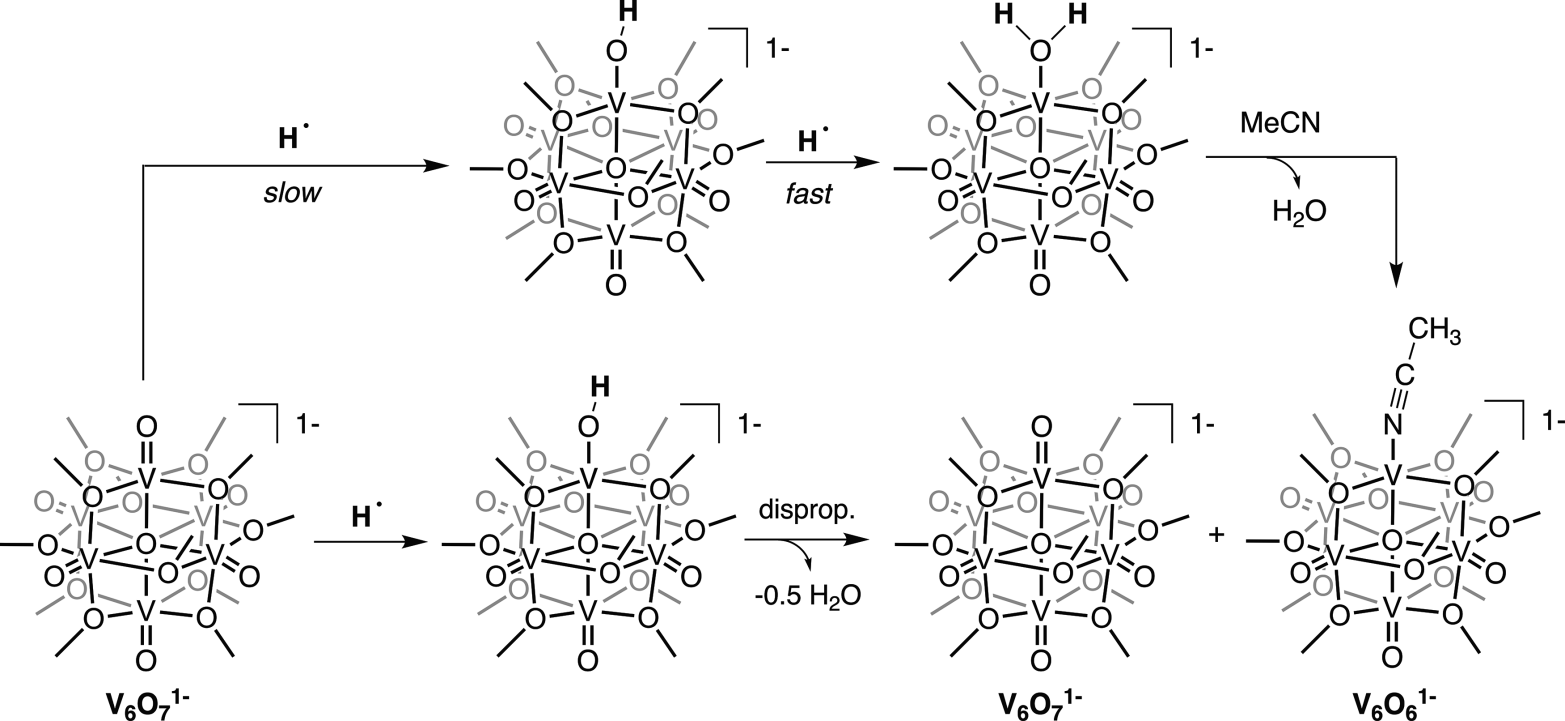
Proposed Mechanisms for the Formation of **2-V**_**6**_**O**_**6**_^**1–**^ via HAT

With this information, we are able to propose two possible reaction mechanisms for the formation of **V**_**6**_**O**_**6**_^**1–**^ (Scheme 3): (1) An initial rate-limiting CPET step forming [V_6_O_6_(OH)(OCH_3_)_12_]^1–^ is followed by the rapid transfer of a second proton–electron pair to generate [V_6_O_6_(OH_2_)(OCH_3_)_12_]^1–^. This multistep PCET reaction is followed by displacement of the aquo ligand from the surface of the cluster by MeCN. (2) An initial rate-limiting CPET step results in the formation of [V_6_O_6_(OH)(OCH_3_)_12_]^1–^, which rapidly disproportionates to form half an equivalent of both the initial cluster, **V**_**6**_**O**_**7**_^**1–**^, and the aquo species. The aquo ligand then dissociates from the cluster, resulting in the formation of **V**_**6**_**O**_**6**_^**1–**^.

Of the aforementioned mechanisms, the second pathway can be eliminated as a possibility due to the observed quantitative formation of **V**_**6**_**O**_**6**_^**1–**^; disproportionation necessitates that some amount of parent POV-alkoxide be present at the completion of the reaction. Further support for mechanism 1 is derived from recent work from our research team describing the reactivity of POV-alkoxide clusters with a silyl radical transfer reagent.^33^ In this study, the addition of half an equivalent of Mashima’s regent (1,4-bis(trimethylsilyl)dihydropyrazine) to **V**_**6**_**O**_**7**_^**1-**^ results in the formation of the isolable siloxide functionalized assembly, [V_6_O_6_(OSiMe_3_)(OMe)_12_]^1-^. Subsequent addition of an equivalent of silyl radical to [V_6_O_6_(OSiMe_3_)(OMe)_12_]^1–^ results in the formation of the oxygen-deficient POV-alkoxide cluster, **V**_**6**_**O**_**6**_^**1–**^, with the concomitant formation of hexamethyldisiloxane. Considering that silyl radicals have recently garnered popularity as substrates that effectively model the reactivity of H-atoms,^34−36^ we hypothesize that HAT for O-atom defect formation follows a similar pathway.

While there have been a few previous studies providing mechanistic insight into the activation of M=O bonds at the surface of POMs and materials through PCET, a significant amount of work has probed the high-valent metal-oxo activation via HAT with monometallic, molecular metal oxide compounds.^22,37−43^ Most often, the reduction of an M=O bond proceeds through a multistep reaction pathway (see mechanism 1, Scheme 3). Comparison of the reactivity between the corresponding M=O and M–OH compounds reveals that the hydroxide species is significantly more reactive, sometimes unobservable by transient spectroscopic analysis. We note here that in a similar manner, no spectroscopic evidence of a hydroxide-containing intermediate has been observed over the course of our studies. Even at early time points at reduced temperatures, the seemingly direct formation of **V**_**6**_**O**_**6**_^**1–**^ is observed following the addition of HAT reagents to **V**_**6**_**O**_**7**_^**1-**^ (Figure S4).

### Charge-State Dependence on H-Atom Uptake in [V_6_O_7_(OCH_3_)_12_]*^n^* (*n* = 0, +1)

Intrigued by defect formation resulting from H-atom uptake in **V**_**6**_**O**_**7**_^**1-**^, we became curious as to whether cluster charge state plays a significant role in the HAT reactivity of the vanadium oxide assembly. In previous work, we have noted a substantial trend between oxidation-state distribution and cluster basicity, identifying that more reduced clusters exhibit a higher affinity for protons. Using the estimated p*K*_a_ values for the corresponding transient hydroxide-substituted POV-alkoxide clusters and reduction potentials for all redox states of the relevant assemblies, we can estimate the theoretical BDFE for a V–OH site in more oxidized congeners (eq 1 and Table 1). We find that this theoretical O–H bond strength increases by ∼2.5 kcal/mol for each equivalent of electrons removed from the cluster core. This trend mirrors observations by Mayer and co-workers, wherein the relative proportion of Ce^III^ and Ce^IV^ centers in colloidal cerium oxide nanoparticles directs the reactivity of H-atom donors, with more oxidized particles featuring improved H-atom abstraction properties over their reduced analogues (*vide infra*).^46^

**Figure 6:**
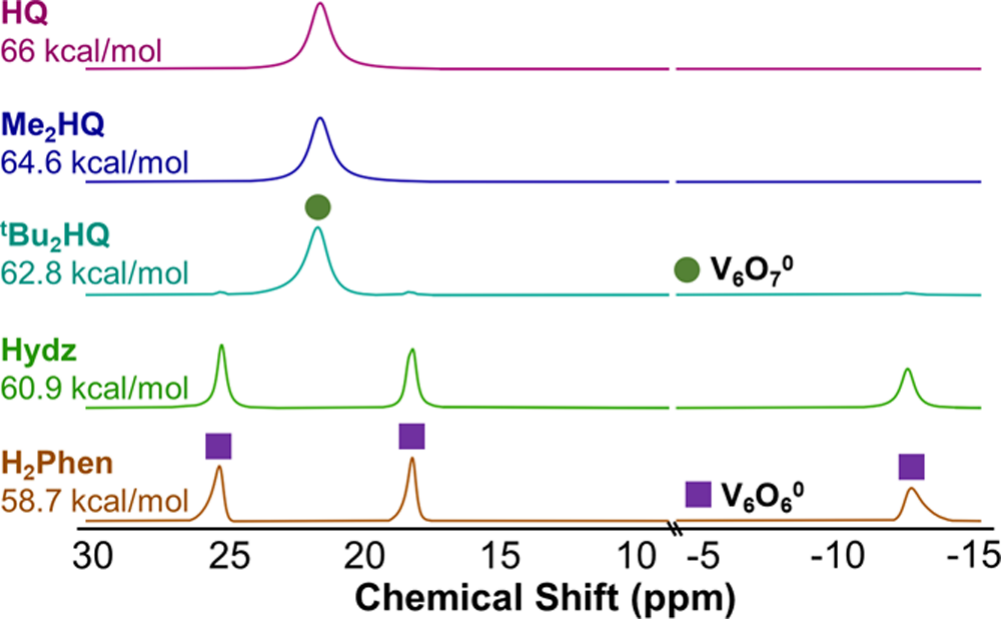
^1^H NMR spectra of reactions between **V**_**6**_**O**_**7**_^**0**^ and 2 e^–^/2 H^+^ donors in CD_3_CN at 21 °C.

To assess whether these expected trends are empirically operative, we next investigated HAT reactivity between the more oxidized forms of the POV-alkoxide (e.g., [V_6_O_7_(OCH_3_)_12_], **V**_**6**_**O**_**7**_^**0**^; [V_6_O_7_(OCH_3_)_12_][OTf], **V**_**6**_**O**_**7**_^**1+**^) and the library of H-atom donors. In the case of complex **V**_**6**_**O**_**7**_^**0**^, analysis of the ^1^H NMR spectrum of the crude reaction mixture following exposure of the cluster to an equivalent of H_2_Phen suggests the quantitative removal of a terminal oxido group (Figure 6). The three paramagnetically shifted and broadened resonances located at 25.3, 18.2, and −12.6 ppm match those previously reported for the neutral O-atom-deficient species, [V_6_O_6_(OCH_3_)_12_(MeCN)] (**V**_**6**_**O**_**6**_^**0**^).^29^ Analogous conversion of the neutral POV-alkoxide to its oxygen-deficient congener was observed following the addition of 1 equiv of Hydz. Time-point analysis reveals quantitative conversion to **V**_**6**_**O**_**6**_^**0**^ after 2 h at room temperature, suggesting improved reactivity of the oxidized form of the cluster over its reduced congener (Figure S5). Indeed, the formation of **V**_**6**_**O**_**6**_^**0**^ on preparatory scales was accomplished via the addition of Hydz to **V**_**6**_**O**_**7**_^**0**^, resulting in isolation of the neutral, oxygen-deficient assembly in good yield (76%). Minor formation of **V**_**6**_**O**_**6**_^**0**^ was observed when **V**_**6**_**O**_**7**_^**0**^ was reacted with ^t^Bu_2_HQ, consistent with the proposed thermodynamic ceiling of reactivity for the neutral cluster (Figure 5 and Table 1). Indeed, upon heating the reaction to 50 °C for 65 h, formation of the neutrally charged, O-atom vacant product, **V**_**6**_**O**_**6**_^**0**^, can be observed by ^1^H NMR spectroscopy (Figure S6).

**Figure 7:**
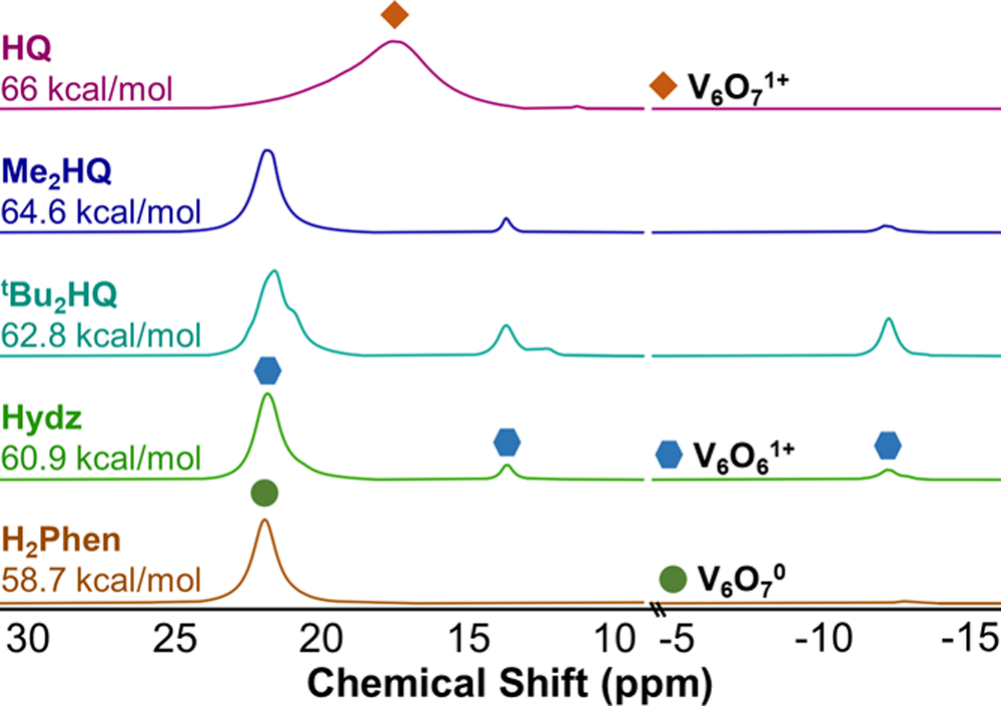
^1^H NMR spectra of reactions between **V**_**6**_**O**_**7**_^**1+**^ and 2e^–^ / 2H^+^ donors in CD_3_CN at 21 °C.

Attempts to evaluate HAT for the formation of O-atom defects at the surface of the cationic POV-alkoxide cluster, **V**_**6**_**O**_**7**_^**1+**^, were complicated by competing for the reduction of the assembly. Upon addition of an equivalent of H_2_Phen to **V**_**6**_**O**_**7**_^**1+**^, rapid formation of **V**_**6**_**O**_**7**_^**0**^ was observed (Figures 7 and S7). The oxidizing nature of complex **V**_**6**_**O**_**7**_^**1+**^ renders electron transfer from this H-atom donor thermodynamically favorable (E_1/2_ H_2_Phen = −0.187 V vs Fc^+/0^ (Figure S8); E_1/2_**V**_**6**_**O**_**7**_^**1+**^ = +0.25 V vs Fc^+/0^); thus, reduction of the cluster to its neutral congener becomes a kinetically competitive side reaction in this system.

On the other hand, reactions with HAT reagents with stronger E–H bonds resulted in the formation of a mixture of species (Figure 7). Exposure of **V**_**6**_**O**_**7**_^**1+**^ to Hydz results in the formation of **V**_**6**_**O**_**7**_^**0**^ as the major project, with a small amount of species with three paramagnetically shifted and broadened resonances located at 21.4, 13.7, and −11.8 ppm. These signals are consistent with those reported for the acetonitrile-bound, oxygen-deficient POV-alkoxide cluster, [V_6_O_6_(OCH_3_)_12_(MeCN)](OTf) (**V**_**6**_**O**_**6**_^**1+**^).^24^ Similar results were observed with Me_2_HQ. We note improved product conversion at early time points using ^t^Bu_2_HQ as the H-atom source; however, in all reactions, the major product observed is complex **V**_**6**_**O**_**7**_^**0**^ (*vide infra*). Reaction of HQ, which features the strongest E–H bonds in the series of H-atom donors investigated here, with **V**_**6**_**O**_**7**_^**1+**^ did not result in any observable reactivity. Notably, the single resonance corresponding to the cationic POV-alkoxide was broadened, likely as a consequence of H-bonding between HQ and a terminal V=O moiety at the cluster surface.

**Figure 8:**
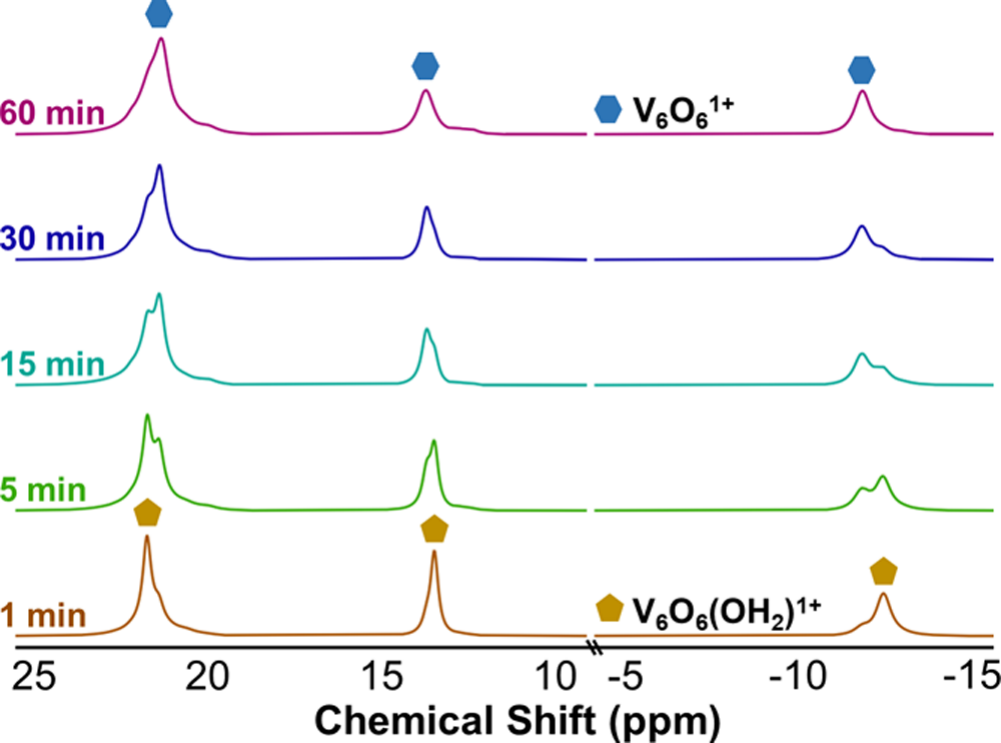
^1^H NMR spectra of reactions between **V**_**6**_**O**_**7**_^**1+**^ and ^t^Bu_2_HQ in CD_3_CN at 21 °C at selected time points.

To gain additional insight into defect formation at the surface of **V**_**6**_**O**_**7**_^**1+**^, we performed *in situ* analysis of HAT between ^t^Bu_2_HQ and the cationic POV-alkoxide cluster (Figure 8). To our surprise, after 1 min, complete conversion of **V**_**6**_**O**_**7**_^**1+**^ to a new product with a distinct three-peak pattern was observed (δ = 21.5, 13.5, −12.4 ppm). In addition, all resonances corresponding to the H-atom donor are consumed at this time point, leaving only signals that indicate the presence of 2,6-di-*tert*-butylbenzoquinone. Being that this species is relatively short-lived, its chemical identity is unknown. However, as the reaction progresses, the resonances corresponding to this product convert to a new set of signals, consistent with the formation of **V**_**6**_**O**_**6**_^**1+**^. Concomitant production of water is observed (Figure S9). These observations suggest that the initial species observed is a POV-alkoxide with a single, terminal V^III^–OH_2_ moiety, which converts to a V^III^–MeCN adduct upon dissociation of H_2_O. However, over time, the integration of the resonance located at ∼21.5 ppm increases disproportionately with those positioned at 13.5 and −12.4 ppm. We pose that this is the result of the formation of **V**_**6**_**O**_**7**_^**0**^ as a byproduct of the reaction, generated as a result of the reaction of **V**_**6**_**O**_**6**_^**1+**^ with water. Indeed, control experiments reveal that the formation of **V**_**6**_**O**_**7**_^**0**^ is observed upon the addition of 1 equiv of water to complex **V**_**6**_**O**_**6**_^**1+**^ (Figure S10).

Unambiguous confirmation of the molecular structure of **V**_**6**_**O**_**6**_^**1+**^ was obtained via single-crystal X-ray diffraction (Figure 3 and Tables 2 and S1). Following refinement of the data, the anticipated product was observed; an acetonitrile solvent molecule is bound to the oxygen-deficient vanadium oxide, with an outer-sphere triflate anion in the unit cell. Broadly speaking, the bond metrics of the Lindqvist ion resemble that of **V**_**6**_**O**_**6**_^**1–**^; a shortened V1–O_c_ bond distance is observed (2.083(3) Å), consistent with the expected truncation of this bond following defect formation. The average V_n_=O_t_ and V_n_–O_c_ bonds (V_n_ = vanadyl ions composing the Lindqvist core) are slightly shorter than those observed in **V**_**6**_**O**_**6**_^**1–**^, consistent with the two-electron oxidation of the cluster core. Notably, all bond distances resemble those reported for the “cationic”, oxygen-deficient POV-alkoxide cluster where the triflate ion is bound to the site-differentiated vanadium center.^28^ Indeed, BVS calculations performed on the six, distinct vanadium ions within the unit cell indicate that the oxidation of vanadyl moieties located *cis* to the vacant site has occurred, in analogy to that observed in the case of [V_6_O_6_(OCH_3_)_12_(OTf)] (Table S3). BVS calculations confirm the proposed oxidation-state distribution of the six vanadium ions as V^III^V_3_^IV^V_2_^V^.

The reactivities of the neutral and cationic POV-alkoxides with organic H-atom donors represent a clear dependence of the oxidation-state distribution of constituent V ions on H-atom abstraction capacity. Our results show that the extraction of electron equivalents from the cluster core results in greater H-atom affinity of a terminal vanadyl site at the surface of the assembly. Based on our experimental findings, we conclude that the BDFE(O–H) of the resultant hydroxide-substituted assemblies, [V_6_O_6_(OH)(OCH_3_)_12_]*^n^* (*n* = 0, 1+), are between 62–63 and 65–66 kcal/mol, respectively (Figures 6 and 7). These ranges are consistent with the calculated BDFE(O–H)s of the transient VO–H bonds formed upon H-atom transfer to the cluster surface in these relevant charge states using the Bordwell equation (Table 2). We again emphasize that these BDFEs are based upon approximated p*K*_a_ values for the protonated species; therefore, the observed reactivities of the POV-alkoxides in the various charge states do not precisely reflect what is predicted using thermodynamic considerations. That said, the empirical and theoretical H-atom affinities are self-consistent.

Although we have seen evidence for oxidation state impacting the driving force of HAT to the polyoxovanadate surface, we believe that the mechanism for the activation of M=O bonds is constant across all charge states of the POV-alkoxide studied here. Evidence can be seen in the reduction of the oxidized cluster **V**_**6**_**O**_**7**_^**0**^ by the deuterated compound D_2_Phen, where a decrease in the observed rate constant results in a KIE value of *k*_obs-H_/*k*_obs-D_ = 2.1 (*k*_obs-H_ = 4.0 × 10^–3^; *k*_obs-D_ = 1.9 × 10^–3^), suggesting that the H-atom is involved in the rate-limiting step to a similar degree in both the oxidized and reduced versions of the cluster (Figure S11). Further evidence supporting our hypothesis of a constant mechanism across charge states was obtained through determination of the fact that activation of a terminal V=O bond in **V**_**6**_**O**_**7**_^**0**^ proceeds first-order with respect to the reductant (Figure S12). We were unable to determine the order of the reaction with respect to cluster. This is due to the fact that in the presence of an extreme excess of reductant, as required under pseudo-first-order reaction conditions, complex **V**_**6**_**O**_**6**_^**0**^ degrades.

Due to the fact that both **V**_**6**_**O**_**7**_^**1–**^ and **V**_**6**_**O**_**7**_^**0**^ are able to react with H_2_Phen to form the respective vacancy product, the impact oxidation state of the cluster has on the reactivity of the assembly can be investigated. The observed rate constants for the reduction of **V**_**6**_**O**_**7**_^**1–**^ and **V**_**6**_**O**_**7**_^**0**^ by H_2_Phen reveal that upon oxidation of the cluster, an increase in the rate constant is observed (Table 3). These results are in agreement with our experimental observation that the oxidized forms of the cluster have a higher H-atom affinity in comparison to reduced variants and suggest that electron density of the cluster has an impact on the ability for reduction to occur at the terminal oxide site.

**Table 3:** Activation Parameters for the Reaction between the POV-Alkoxide Cluster at Various Oxidation States and the Reductant Dihydrophenazine^a^

cluster	*k*_obs-H_(s^–1^)	*k*_obs-D_(s^–1^)	Δ*H*^‡^ (kcal mol^–1^)	Δ*S*^‡^ (cal mol^–1^ K^–1^)	ΔG^‡^ (kcal mol^–1^)
**V**_**6**_**O**_**7**_^**1–**^	1.0 × 10^–3^	5.0 × 10^–4^	6.5 ± 0.8	–40.9 ± 3.1	18.7 ± 1.7
**V**_**6**_**O**_**7**_^**0**^	4.0 × 10^–3^	1.9 × 10^–3^	7.8 ± 0.8	–31.0 ± 3.3	17.1 ± 1.8

a Values were obtained from the Eyring plots in Figure 9 for **V**_**6**_**O**_**7**_^**1–**^ and **V**_**6**_**O**_**7**_^**0**^. ΔG^‡^ is reported for a temperature of 298 K.

Further details into the impact oxidation state imparts on the ability to perform HAT can be obtained through measuring the temperature dependence on the observed rate constants for the reduction of both **V**_**6**_**O**_**7**_^**1–**^ and **V**_**6**_**O**_**7**_^**0**^. Eyring plots were obtained by varying the temperature of the reaction while measuring the rate of formation of the respective vacancy product, allowing for the determination of activation parameters such as activation enthalpy, Δ*H*^‡^, entropy, Δ*S*^‡^, and free energy ΔG^‡^. For the reduction of **V**_**6**_**O**_**7**_^**1–**^ by H_2_Phen (Figure 9), we obtain the parameters of Δ*H*^‡^ = 6.5 ± 0.8 kcal mol^–1^and Δ*S*^‡^ = −40.9 ± 3 cal mol^–1^ K^–1^. The large, negative value of Δ*S*^‡^ indicates a bimolecular reaction, where a single, well-ordered transition state is formed during the rate-limiting step.

**Figure 9:**
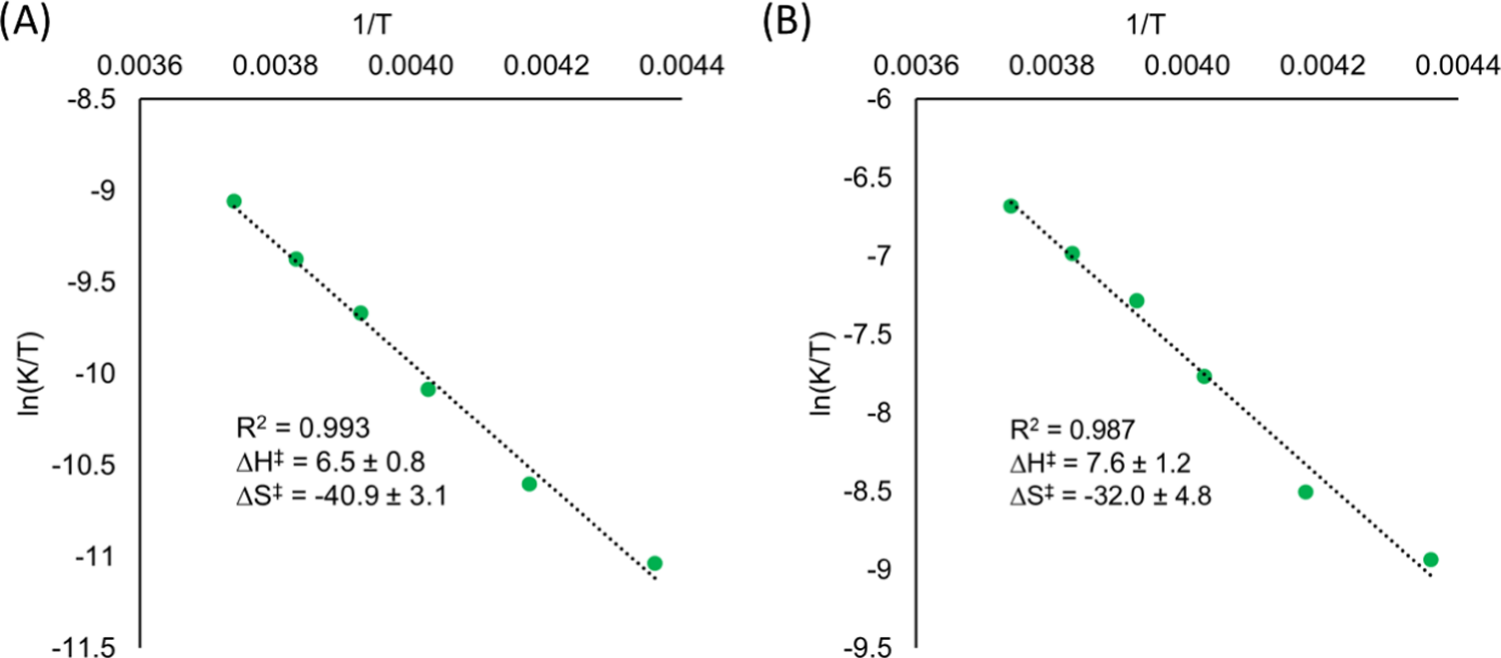
Eyring plot for the reaction between (A) **V**_**6**_**O**_**7**_^**1–**^ and (B) **V**_**6**_**O**_**7**_^**0**^ and H_2_Phen. Reactions were run in pseudo-first-order conditions, where the concentration of the cluster was in excess at 5 mM, while the reductant was at 1 mM. The rate of reaction was measured across a range of temperatures from −43 to −5 °C. Values along the *Y*-axis were found by dividing the observed rate constant, *k*_obs_, by the concentration of the respective cluster in excess to obtain the rate constant *K*.

Previous studies have reported that a relatively small activation enthalpy Δ*H*^‡^ value combined with the large negative activation entropy Δ*S*^‡^ serves as additional evidence for CPET mechanisms.^44,45^ Because CPET is an inner-sphere process, reorganization is required in order for the reductant to make van der Waals contact with the terminal oxido site, suggesting that the entropy term will contribute significantly to the total activation energy. This observation provides additional support for the activation of terminal vanadyl moieties via CPET.

The construction of an Eyring plot for the reaction between **V**_**6**_**O**_**7**_^**0**^ and H_2_Phen allows for the activation parameters to be directly compared to the monoanionic cluster (Figure 9 and Table 3). Results from these experiments reveal that the activation free energy for the reduction of the neutral cluster **V**_**6**_**O**_**7**_^**0**^ is approximately 1.6 kcal mol^–1^ smaller as compared to that of **V**_**6**_**O**_**7**_^**1–**^. This would agree with the observation that upon oxidation, the POV cluster’s affinity toward H-atoms increases. Comparing the values for the enthalpy of activation reveals an increase in the energy required to reach the activated transition complex upon the oxidation of **V**_**6**_**O**_**7**_^**1–**^ to the neutral cluster, **V**_**6**_**O**_**7**_^**0**^. In order for PCET to occur through a CPET pathway, preorganization of a donor–acceptor pair must occur through the formation of a hydrogen bond. Due to the fact that the monoanionic cluster has a greater density of charge, the terminal metal oxide ligands are more basic, resulting in conditions more favorable for hydrogen bonding to occur.

## Conclusions

Here, we present the activation of terminal M=O bonds at the surface of metal oxide clusters through proton-coupled electron transfer as a function of molecular charge state. By introducing H-atom donors to the fully oxygenated assembly, we can facilitate the quantitative formation of an oxygen-deficient species. This improved preparative pathway has allowed for isolation and structural analysis of previously unattainable vacancy products, such as **V**_**6**_**O**_**7**_^**1–**^ and **V**_**6**_**O**_**6**_^**1+**^. The observed reactivity is reminiscent of H-atom uptake in solid-state vanadium oxides, providing insight into the products of hydrogen incorporation into extended materials. Kinetic analysis of defect formation suggests that the V=O bond cleavage occurs via CPET; our proposed reaction pathway includes an initial rate-limiting step of CPET to the terminal oxo site, followed by a rapid transfer of a second H-atom equivalent. Displacement of the water ligand by acetonitrile then results in the formation of the O-atom vacancy cluster. We hypothesize that this general reaction mechanism of the V=O bond cleavage via HAT is retained across all charge states of the assembly.

Further analysis into the reactivity of POV-alkoxide clusters toward M=O activation via HAT reveals a trend in the ability to extract a H-atom from the substrate based on the oxidation state of the cluster. Both thermochemical and kinetic analyses reveal that as electron density of the cluster decreases, the affinity toward H-atom abstraction increases. While this phenomenon has yet to be explored with polyoxometalates, Agapie and co-workers have demonstrated that the BDFE(O–H) of a terminal M–OH moiety (M = Mn, Fe) embedded in an iron oxide cluster is likewise tuned by the oxidation-state distribution of distal iron centers.^20,21^ Notably, the BDFE(O–H) proposed for the transient hydroxide species formed *en route* to the V=O bond cleavage in this work is substantially weaker (BDFE(V–OH) = 61–66 kcal/mol) than that reported for the metal hydroxides described above (BDFE(M–OH) = 72–84 kcal/mol, M = Fe; 92–104 kcal/mol, M = Mn).

Critically, these periodic trends lend insight to design criteria for nanoscale metal oxide materials with targeted HAT reactivity of relevance to small-molecule activation. Indeed, recent work from the Mayer group has reported that the percentage of reduced metal sites at the surface of ceria nanoparticles has a direct impact on the ability to form hydroxide ligands through PCET.^46^ In comparing the results from the ceria nanoparticles and the POV-alkoxides studied here, neither example appears to follow shifts in BDFE(O–H) predicted by the Nernst equation (in the case of the clusters studied here, the Nernst equation predicts a decrease of ∼0.2 kcal/mol per electron added). In fact, both examples result in a change of BDFE(O–H), an order of magnitude larger than would be expected. One explanation proposed by the Mayer group suggests that a distribution of chemically distinct sites exists at the surface of the metal oxide nanoparticle. These sites likely occur as a result of charge localization at metal centers, inducing changes in the M–O bond lengths and altering the ligand preferences for a particular site. POVs are typically described as Robin and Day class II delocalized systems; crystal structure analysis reveals that partial electron localization can be observed in these assemblies, as noted in the differentiation of V^V^/V^IV^ centers through variations in V–O bond lengths. This translates to a proposal that chemically distinct sites may exist at the surface of POV-alkoxides. Oxidation of the cluster alters this distribution, favoring the abstraction of H-atoms more so than what would be expected by the Nernst equation alone. In the case of the work from the Agapie group described above, the authors observe significantly larger changes in BDFE(O–H) than would be predicted.^20,21^ These changes in reactivity of the terminal M=O bond more closely resemble those reported mononuclear metal oxidos, as opposed to extended metal oxide nanostructures.

Collectively, the thermochemical and kinetic analyses of PCET at the surface of POV-alkoxide clusters have presented insight into a novel form of V=O bond activation at polyoxometalate surfaces. In addition, the comparison of reactivity across a range of oxidation states establishes trends that allow for reactivity toward HAT to be predicted. Ongoing efforts in our laboratory include probing the impact surface chemistry of polyoxometalates imparts on the ability to perform H-atom abstraction and how this information can impact the design of systems in which the transfer of both electrons and protons is required.

## Experimental Section

### General Considerations

All manipulations were carried out in the absence of water and oxygen using standard Schlenk techniques or in a UniLab MBraun inert atmosphere drybox under a dinitrogen atmosphere. All glassware was oven-dried for a minimum of 4 h and cooled in an evacuated antechamber prior to use in the drybox. Solvents were dried and deoxygenated on a glass contour system (Pure Process Technology, LLC) and stored over 3 Å molecular sieves purchased from Fisher Scientific and activated prior to use. 2,6-Dimethyl-1,4-hydroquinone was purchased from TCI America and used as received. 2.5 M *n*-Butyllithium in hexanes was purchased from Sigma-Aldrich and used as received. D_2_O was purchased from Cambridge Isotope Laboratories and used as received. POV-alkoxide clusters **V**_**6**_**O**_**7**_^**1-**^, **V**_**6**_**O**_**7**_^**0**^, and **V**_**6**_**O**_**7**_^**1+**^ were prepared according to previously reported procedures.^24,47,48^ 2,6-Di-*tert*-butyl-1,4-hydroquinone,^27^ 1,4-hydroquinone,^27^ and 5,10-dihydrophenazine^49^ were generated following literature precedent.

^1^H NMR spectra were recorded at 400 MHz or 500 MHz on a Bruker DPX-400 or Bruker DPX-500 spectrometer, respectively, locked on the signal of deuterated solvents. All chemical shifts were reported relative to the peak of the residual H signal in deuterated solvents. CD_3_CN was purchased from Cambridge Isotope Laboratories, degassed by three freeze–pump–thaw cycles, and stored over fully activated 3 Å molecular sieves.

Single crystals of [*^n^*Bu_4_N][V_6_O_6_(MeCN)(OCH_3_)_12_] (**V**_**6**_**O**_**6**_^**1–**^) and [V_6_O_6_(MeCN)(OCH_3_)_12_][OTf] (**V**_**6**_**O**_**6**_^**1+**^) were mounted on the tip of a thin glass optical fiber (goniometer head) and on an XtaLab Synergy-S Dualflex diffractometer equipped with a HyPix-6000HE HPC area detector for data collection at 100.00(10)–192.99(10) K, respectively. The structures were solved using SHELXT-2018/2^50^ and refined using SHELXL-2018/3.^51^

#### Synthesis of [*^n^*Bu_4_N][V_6_O_6_(OCH_3_)_12_(MeCN)] (**V**_**6**_**O**_**6**_^**1–**^)

A 20 mL scintillation vial was charged with **V**_**6**_**O**_**7**_^**1-**^ (0.056 g, 0.055 mmol), a stir bar, and 6 mL of MeCN. In a separate vial, 1 equiv of 5,10-dihydrophenazine (0.010 g, 0.055 mmol) was dissolved in 4 mL of MeCN. The second solution was added dropwise to the cluster solution with vigorous stirring. The reaction solution was stirred for 1 h, over which time the color changed from green to red-brown. Next, solvents were removed under reduced pressure, leaving a brown-red residue. The crude reaction mixture was washed with *n*-pentane (3 × 10 mL) and then once with 10 mL of diethyl ether. The remaining solid was extracted in MeCN and dried *in vacuo* to yield [*^n^*Bu_4_N][V_6_O_6_(MeCN)(OCH_3_)_12_] **V**_**6**_**O**_**6**_^**1–**^ (0.055 g, 0.052 mmol, 95%). Crystals suitable for analysis via ^1^H NMR spectroscopy were produced by slow evaporation of the extraction solution. Formation and purity of **V**_**6**_**O**_**6**_^**1–**^ were confirmed by ^1^H NMR spectroscopy; paramagnetically shifted and broadened resonances consistent with those reported previously by our research group were observed.^28^

#### Synthesis of [V_6_O_6_(OCH_3_)_12_(MeCN)] (**V**_**6**_**O**_**6**_^**0**^)

A 20 mL scintillation vial was charged with **V**_**6**_**O**_**7**_^**0**^ (0.053 g, 0.067 mmol), a stir bar, and 6 mL of MeCN. In a separate vial, 1 equiv of hydrazobenzene (0.012 g, 0.067 mmol) was dissolved in 4 mL of MeCN. The second solution was added dropwise to the cluster solution with vigorous stirring. The reaction solution was stirred for 1 h, over which time the color changed from green to brown, and then it was dried *in vacuo*. The crude mixture was washed with *n*-pentane (3 × 10 mL) and then once with 10 mL of diethyl ether. The remaining solid was extracted in MeCN and dried *in vacuo* to yield **V**_**6**_**O**_**6**_^**0**^ (0.042 g, 0.052 mmol, 77%). Formation and purity of **V**_**6**_**O**_**6**_^**0**^ were confirmed by ^1^H NMR spectroscopy; paramagnetically shifted and broadened resonances consistent with those reported previously by our research group were observed.^29^

#### Synthesis of [V_6_O_6_(OCH_3_)_12_(MeCN)][OTf] (**V**_**6**_**O**_**6**_^**1+**^)

A 20 mL scintillation vial was charged with **V**_**6**_**O**_**7**_^**1+**^ (0.053 g, 0.057 mmol) with a stir bar and dissolved in 6 mL of MeCN. In a separate vial, 1 equiv of 2,6-di-*tert*-butyl-1,4-hydroquinone (0.013 g, 0.057) was dissolved in 4 mL of MeCN. Both solutions were frozen in a liquid N_2_-chilled cold well. Both vials were removed from the cold well, and while thawing and stirring, one-third of the HAT reagent solution was added to the cluster containing vial, stirred until fully thawed, and returned to the cold well. This was repeated two times, until all of the HAT reagent had been added to the cluster solution. This solution was stirred for 1 h, after which time solvents were removed under reduced pressure. Subsequently, the product was dissolved in 1 mL of THF with 1 drop of MeCN and crystallized by slow evaporation of pentane into the THF solution to yield [V_6_O_6_(OCH_3_)_12_(MeCN)][OTf]·THF (**V**_**6**_**O**_**6**_^**1+**^). Drying of the crystals *in vacuo* removed the cocrystallized THF for elemental analysis. ^1^H NMR (500 MHz, CD_3_CN): δ = 21.36, 13.71, −11.81 ppm. Elemental analysis for C_15_H_39_NO_21_SF_3_V_6_ (MW: 964.17 g/mol) Calc’d (%): C, 18.69; H, 4.08; N, 1.45. Found (%): C, 18.747; H, 3.861; N, 1.349.

#### General Procedure for Time-Point Analysis of H-Atom Abstraction Reactivity of POV-Alkoxides with Organic H-Atom Donors

A J. Young tube was charged with a sample of POV-alkoxide cluster (∼0.030 g) dissolved in ∼0.5 mL CD_3_CN. The solution was frozen in the tube in a cold well cooled with liquid N_2_. In a vial, 0.5 or 1 equiv of organic H-atom donor was dissolved in ∼0.5 mL of CD_3_CN. Once the cluster solution was frozen, the H-atom donor solution was added to the J. Young tube and frozen in the cold well. When the solutions were frozen solid, the tube was sealed, quickly removed from the glovebox, and stored over dry ice before analysis. When ready for analysis, the solution was thawed and inserted into an NMR spectrometer. Subsequent analysis of reaction progress was performed at regular intervals at 25 °C until the spectrum ceased to evolve.

#### General Procedure for Performing Pseudo-First-Order Reaction Kinetics

Pseudo-first-order reaction conditions were used to establish the rate expression for the reaction between the POV-alkoxide cluster, [V_6_O_7_]*^n^* (where *n* = 1–, 0) and a H-atom transfer reagent, 5,10-dihydrophenazine or hydrazobenzene. To determine the order of each reactant with respect to the rate expression, the initial rate of the reaction was measured using ^1^H NMR spectroscopy, where the concentration of the product cluster, [V_6_O_6_]*^n^*, can be measured over time. To find the order with respect to the cluster, a 0.4 mL sample of acetonitrile-*d*_3_ (CD_3_CN) containing 6.25 mM of the desired HAT reagent and 2.5 mM hexamethyldisiloxane (HMDS) as an internal standard was prepared in a J-Young tube. The sample was then frozen using liquid nitrogen, and an aliquot of a stock solution of CD_3_CN containing the cluster (5 mM stock solution) was added and kept frozen. If needed, additional CD_3_CN was then added to reach a final volume of 0.5 mL. Once frozen, the sample was quickly transferred to the spectrometer set to the desired temperature. A ^1^H NMR spectrum was then collected every 10 s for 8 min to collect the initial rate of reaction. Once completed, the reaction was repeated using a different concentration of [V_6_O_7_]*^n^*.

To establish the order with respect to the HAT reagent, a 0.4 mL sample of CD_3_CN containing 6.25 mM cluster and 2.5 mM HMDS as an internal standard was prepared in a J-Young tube. The sample was then frozen using liquid nitrogen, and an aliquot of a stock solution of CD_3_CN containing the HAT reagent (5 mM stock solution) was added and kept frozen. If needed, additional CD_3_CN was then added to reach a final volume of 0.5 mL. Once frozen, the sample was quickly transferred to the spectrometer set to the desired temperature. A ^1^H NMR spectrum was then collected every 10 s for 8 min to collect the initial rate of reaction. Once completed, the experiment was repeated with a different volume of the HAT reagent stock solution.

#### General Procedure for Establishing Pseudo-First-Order Rate Constants for the Reduction [V_6_O_7_]*^n^* by HAT

Rate constants were determined using kinetic data obtained by measuring the initial rate of formation for the product cluster, [V_6_O_6_]*^n^*, over a range of concentrations of the H-atom transfer reagent (HAT). The general rate law for the reaction between the POV-alkoxide cluster [V_6_O_7_]*^n^* (where *n* = 1–, 0) and a H-atom transfer reagent (5,10-dihydrophenazine or hydrazobenzene) can be seen in eq 3. To compare the rate constants between different oxidation states of the cluster, pseudo-first-order reaction conditions were used, in which the concentration of the cluster was held in excess compared to that of the HAT reagent. As a result of these conditions, the rate law can be simplified to eq 4
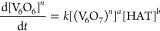
3
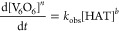
4
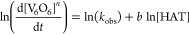
5where [HAT] represents the initial concentration of the H-atom transfer reagent. Plotting the natural log of the initial rate of reaction vs the natural log of the concentration of the HAT reagent (eq 5) allows for the pseudo-first-order rate constant to be determined. The observed rate constant can be extracted from the *y*-intercept of this plot. From the slope of this graph, we can determine the order with respect to the HAT reagent to be 1, indicating that the units of the observed rate constant are s^–1^.

#### General Procedure for Determining Activation Energy for the Reduction of [V_6_O_7_]*^n^* by HAT

Activation parameters were determined using kinetic data obtained by measuring the initial rate of formation of the product cluster, [V_6_O_6_]*^n^*, over a range of temperatures. Pseudo-first-order reaction conditions were utilized to simplify determining the observed rate constants for each temperature, where the concentration of the cluster was held in excess over the HAT reagent. From the results collected in the variable temperature experiments, the activation parameters are able to be established using the linear form of the Eyring–Polanyi equation shown in eq 6
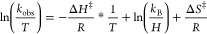
6
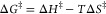
7where *T* is the temperature, Δ*H*^‡^ is the enthalpy of activation, Δ*S*^‡^ is the entropy of activation, *R* is the universal gas constant (*R* = 1.987x^–3^ kcal K^–1^ mol^–1^), *k*_B_ is the Boltzmann constant, and *h* is Planck’s constant. Plotting ln(*k*_obs_/*T*) vs 1/*T* gives a plot with a linear best-fit line, from which the enthalpy of activation can be found by slope = −Δ*H*^‡^/*R*. In addition, the entropy of activation can be found from the *y*-intercept, where *y*-intercept = ln(*k*_B_/*h*) + Δ*S*^‡^/R. From these parameters, the activation free energy can be determined at the desired temperature using eq 7.

#### Synthesis of d_2_-5,10-Dihydrophenazine

A 50 mL round-bottom Schlenk flask was charged with 5,10-dihydrophenazine (0.213 g, 1.17 mmol) and 10 mL of tetrahydrofuran (THF). A solution of 2.5 M *n*-butyllithium in hexanes (0.95 mL, 2.37 mmol) was added dropwise with stirring, where a yellow solid quickly precipitated out of solution. The reaction was stirred at room temperature for 18 h. Volatiles were removed under vacuum, leaving a yellow solid. The reaction vessel was then cooled to 0 °C in an ice bath, whereupon D_2_O (10 mL) was added under a nitrogen flow to give a white precipitate. The reaction was stirred at room temperature for 2 h. The solvent was then removed under vacuum to yield a white solid. The product was extracted with THF (15 mL) and concentrated down to 0.5 mL. Vapor diffusion of pentane into the THF solution affords white, flaky crystals of *d*_2_-5,10-dihydrophenazine (0.063 g, 0.34 mmol, 29%). ^1^H NMR of the crystallized product reveals 92.5% deuteration of the product. ^1^H NMR (400 MHz, CD_3_CN) *d* = 6.12 (m, 4H), 6.39 (m, 4H).

## Supporting Information Available

^1^H NMR spectra of the reaction mixture of POV-alkoxide clusters and HAT reagents, as well as ^1^H NMR spectra of the reaction between **V**_**6**_**O**_**6**_^**1+**^ and water, crystallographic parameters and bond valence sum calculations of complexes **V**_**6**_**O**_**6**_^**1–**^ and **V**_**6**_**O**_**6**_^**1+**^, kinetic analysis of the reaction between POV clusters and HAT reagents, and cyclic voltammogram of dihydrophenazine; crystallographic information files for [*^n^*Bu_4_N][V_6_O_6_(OCH_3_)_12_(MeCN)] (MATES07) [V_6_O_6_(OCH_3_)_12_(MeCN)][OTf] (MATES06) (PDF)

## Accession Codes

CCDC 2130169–2130170 contain the supplementary crystallographic data for this paper. These data can be obtained free of charge via www.ccdc.cam.ac.uk/data_request/cif, or by emailing data_request@ccdc.cam.ac.uk, or by contacting The Cambridge Crystallographic Data Centre, 12 Union Road, Cambridge CB2 1EZ, UK; fax: +44 1223 336033.
