# Supplementary material for: Oxygen-Atom Defect Formation in Polyoxovanadate Clusters via Proton-Coupled Electron Transfer
Source: J Am Chem Soc. 2022 Mar 11;144(11):5029–41. doi: 10.1021/jacs.1c13432 (PMC8949770; doi:10.1021/jacs.1c13432)
Supplement: Supplementary file 1 — ja1c13432_si_001.pdf [file ja1c13432_si_001.pdf]

Electronic Supporting Information

Oxygen-atom defect formation in Polyoxovanadate Clusters via Proton Coupled Electron Transfer

Eric Schreiber†, Alex A. Fertig†, William W. Brennessel, and Ellen M. Matson*
Department of Chemistry, University of Rochester, Rochester NY, USA 14627
† Authors contributed equally to this work

Supporting Information Table of Contents:

Table S1. Crystallographic parameters of the molecular structures obtained for complexes $\mathbf{V_6O_6^{1-}}$ and $\mathbf{V_6O_6^{1+}}$........S2
Figure S1. Diamagnetic region of the ^{1}H NMR spectra of $\mathbf{V_6O_7^{1-}}$ + H_2Phen in CD_3CN at 21 °C........S3
Table S2. Bond valence sum calculations for $\mathbf{V_6O_6^{1-}}$........S3
Figure S2. ^{1}H NMR spectrum of $\mathbf{V_6O_7^{1-}}$ + Hydz after 72 h at 50 °C........S4
Figure S3. Rate comparison for reduction of $\mathbf{V_6O_7^{1-}}$ by H_2Phen and D_2Phen........S4
Figure S4. Low temperature, early time point ^{1}H NMR spectra of the reaction between $\mathbf{V_6O_7^{1-}}$ and H_2Phen........S5
Figure S5. Timepoint analysis of the paramagnetic (left) and diamagnetic (right) regions of the ^{1}H NMR spectra of $\mathbf{V_6O_7^{0}}$ with Hydz in CD_3CN at 21 °C........S5
Figure S6. ^{1}H NMR spectrum of $\mathbf{V_6O_7^{0}}$ + $^tBu_2$HQ after 65 h at 50 °C........S6
Figure S7. Timepoint analysis of the ^{1}H NMR spectra of $\mathbf{V_6O_7^{1+}}$ with H_2Phen in CD_3CN at 21 °C........S6
Figure S8. Cyclic Voltammogram of H_2Phen in MeCN with 0.1 M [$^nBu_4$N][PF_6] supporting electrolyte........S7
Figure S9. Timepoint analysis of the diamagnetic region of the ^{1}H NMR spectra of $\mathbf{V_6O_7^{1+}}$ with $^tBu_2$HQ in CD_3CN........S7
Figure S10. ^{1}H NMR spectrum of $[V_6O_6(OCH_3)_{12}]^{OTf}$ before and after reaction with degassed D_2O in CD_3CN at 21 °C........S8
Table S3. Bond valence sum calculations for $\mathbf{V_6O_6^{1+}}$**.**........S8
Figure S11. Rate comparison for reduction of $\mathbf{V_6O_7^{0}}$ by H_2Phen and D_2Phen........S9
Figure S12. Plot of ln(rate) vs. ln[H_2Phen] to determine the order of H_2Phen in the reaction of $\mathbf{V_6O_7^{0}}$ and H_2Phen........S9

Table S1. Crystallographic parameters for molecular structures of complexes $\mathbf{V_6O_6^{1-}}$ and $\mathbf{V_6O_6^{1+}}$.

Compound	$\mathbf{V_6O_6^{1-}}$	$\mathbf{V_6O_6^{1+}}$
Empirical formula	$C_{30}H_{75}N_2O_{18}V_6$	$C_{19}H_{47}F_3NO_{22}SV_6$
Formula weight	1057.56	1036.27
Temperature / K	99.99(10)	100.00(10)
Wavelength / Å	1.54184	1.54184
Crystal group	Orthorhombic	Monoclinic
Space group	*Pbca*	$P2_1/c$
Unit cell dimensions	*a* =17.99484(19) Å<br>*b* =28.9255(4) Å<br>*c* =36.3322(4) Å<br>α = 90°<br>β = 90°<br>γ = 90°	*a* =19.1402(4) Å<br>*b* =22.0382(4) Å<br>*c* =20.3460(4) Å<br>α = 90°<br>β = 117.216(3)°<br>γ = 90°
Volume / Å^3	18911.3(4)	7632.1(3)
Z	16	8
Reflections collected	124947	95852
Independent reflections	19879	16064
Completeness (theta)	99.9% (67.684°)	99.8% (74.504°)
Goodness-of-fit on F^2	1.022	1.090
Final *R* indices [*I*>2sigma(*I*)]	*R*1 = 0.1304	*R*1 = 0.0713
Largest diff. peak and hole	0.875 and -0.583 e.Å^{-3}	0.655 and -1.221 e.Å^{-3}

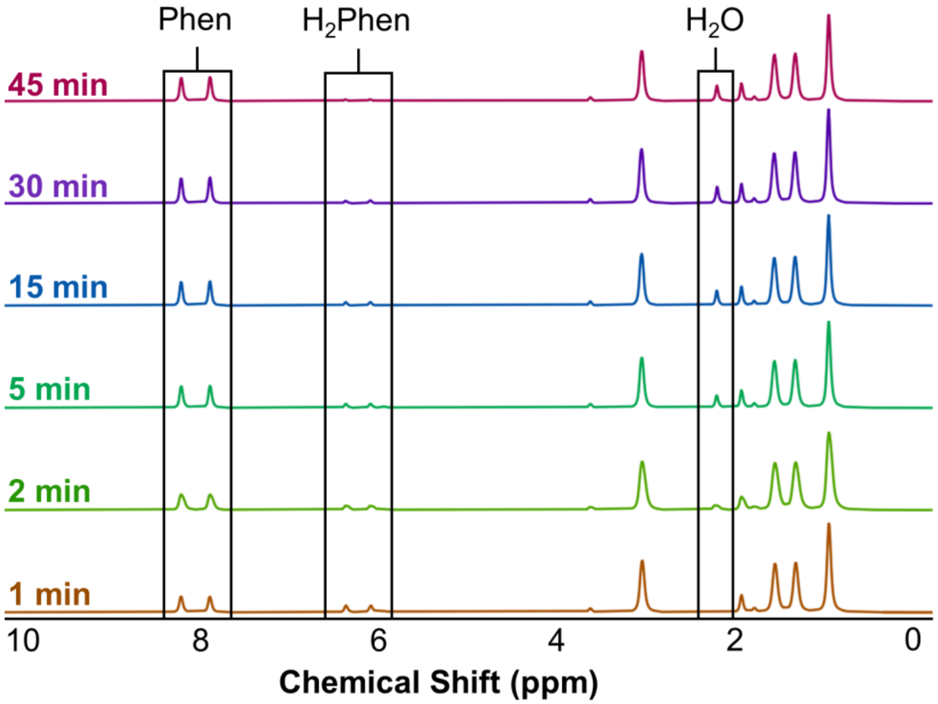

Figure S1. Diamagnetic region of the ^{1}H NMR spectra of $\mathbf{V_6O_7}^{1-}$ + H_2Phen in CD_3CN at 21 °C.

Table S2. Bond valence sum calculations for $\mathbf{V_6O_6}^{1-}$ based on X-Ray crystallographic data collected at 100 K. Table reflects the results of BVS calculations using V-E (E = O, N) valence parameters (r_0) for different oxidation states of vanadium.

$\mathbf{V_6O_6}^{1-}$	V1	V2	V3	V4	V5	V6
V(III)	**3.137**	3.982	3.968	3.885	3.942	3.997
V(IV)	3.212	**4.077**	**4.063**	**3.978**	**4.036**	**4.093**
V(V)	3.451	4.346	4.332	4.243	4.302	4.362

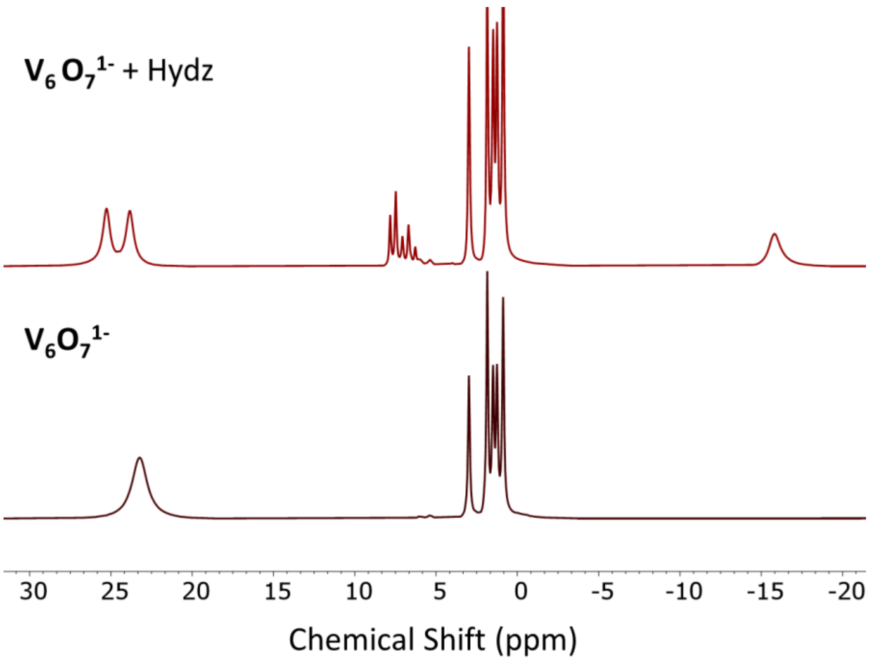

Figure S2. ^{1}H NMR spectra of the cluster $\mathbf{V_6O_7^{1-}}$ (bottom), and the crude spectrum of the reaction between $\mathbf{V_6O_7^{1-}}$ and hydrazobenzene after stirring at 50° C for 72 hours. The characteristic three peak pattern of the anionic, O-atom vacancy product, $\mathbf{V_6O_6^{1-}}$, can be observed in the crude reaction mixture, suggesting the reaction contains a high activation barrier.

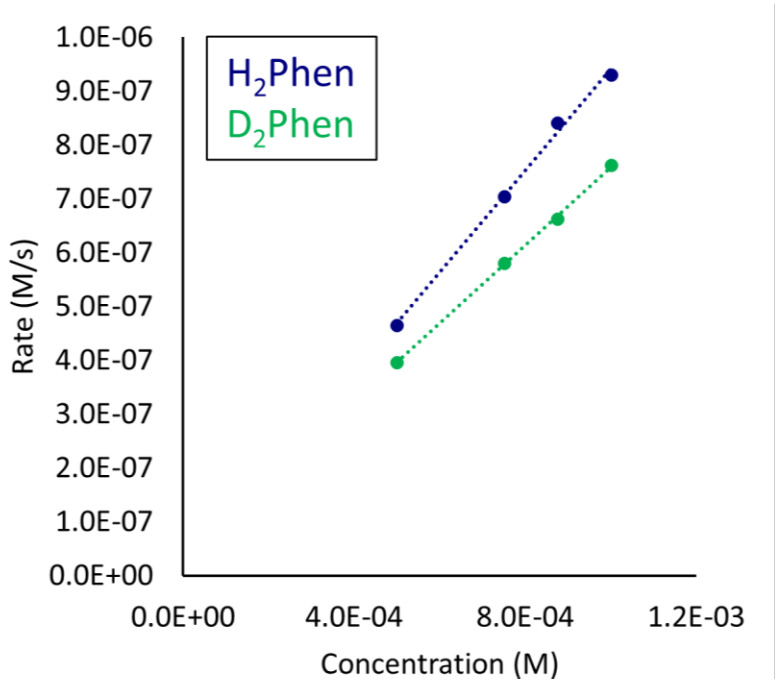

Figure S3. Comparison of the rate of reaction between the monoanionic cluster, $\mathbf{V_6O_7^{1-}}$, and the reductants H_2Phen (blue) and the deuterated version, D_2Phen (green). Results show a decrease in the rate of reaction upon isotopic substitution, suggesting the hydrogen atom is involved in the rate limiting step. All results were collected by measuring rate of formation of the product, $\mathbf{V_6O_6^{1-}}$, using ^{1}H NMR spectroscopy. All experiments were performed in CD_3CN at 0 °C.

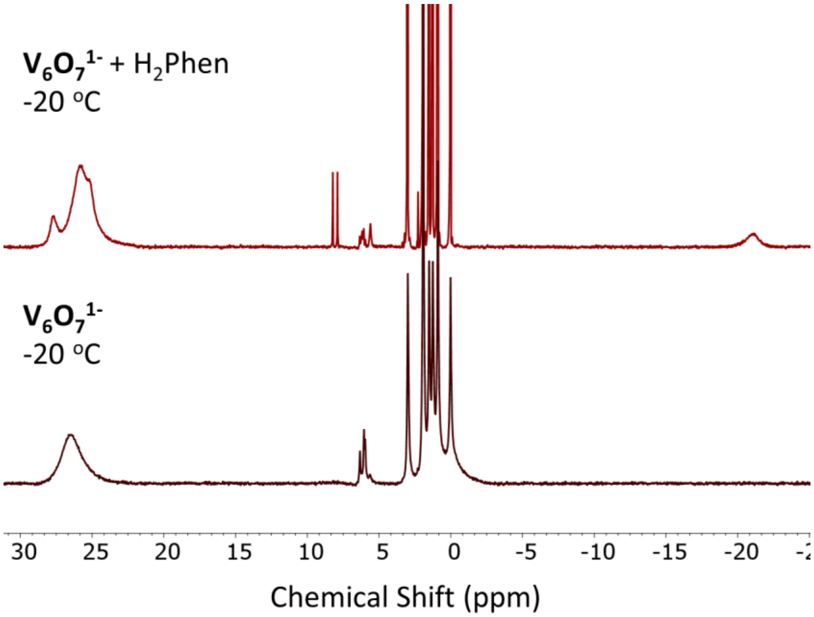

Figure S4. Comparison of the ^{1}H NMR spectra of $\mathbf{V_6O_7^{1-}}$ at -20 °C (bottom), and the crude reaction mixture of $\mathbf{V_6O_7^{1-}}$ and H_2Phen after 30 minutes at -20 °C (top). The formation of the three peak pattern that matches the vacancy product, $\mathbf{V_6O_6^{1-}}$, indicates that no intermediate is able to be seen spectroscopically in this reaction.

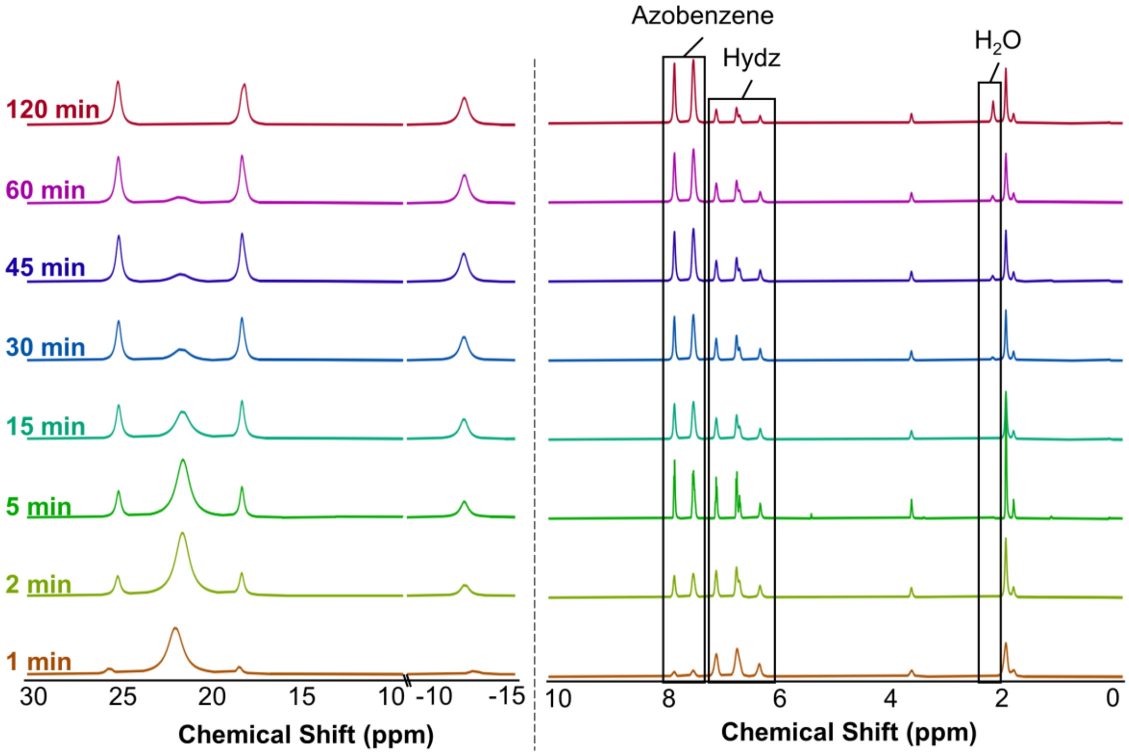

Figure S5. Timepoint analysis of the paramagnetic (left) and diamagnetic (right) regions of the ^{1}H NMR spectra of $\mathbf{V_6O_7^0}$ with Hydz in CD_3CN at 21 °C.

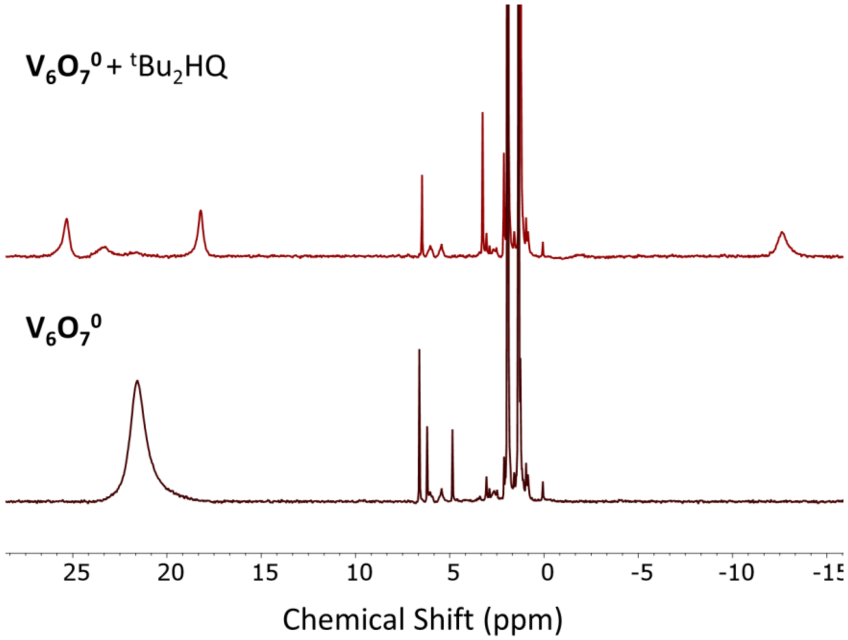

Figure S6. ^1H NMR spectra of the cluster $\mathbf{V_6O_7^0}$ (bottom), and the crude spectrum of the reaction between $\mathbf{V_6O_7^0}$ and $^tBu_2HQ$ after stirring at 50° C for 65 hours. The characteristic three peak pattern of the anionic, O-atom vacancy product, $\mathbf{V_6O_6^0}$, can be observed in the crude reaction mixture, suggesting the reaction contains a high activation barrier.

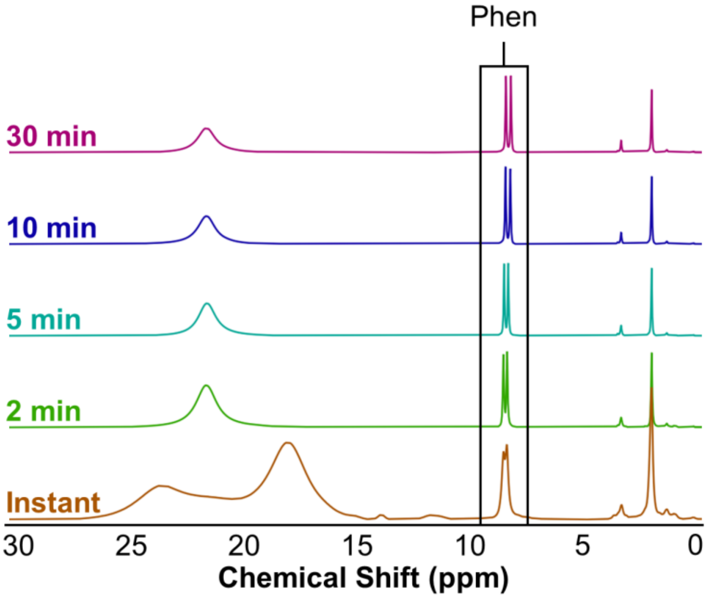

Figure S7. Timepoint analysis of the ^1H NMR spectra of $\mathbf{V_6O_7^{1+}}$ with H_2Phen in CD_3CN at 21 °C.

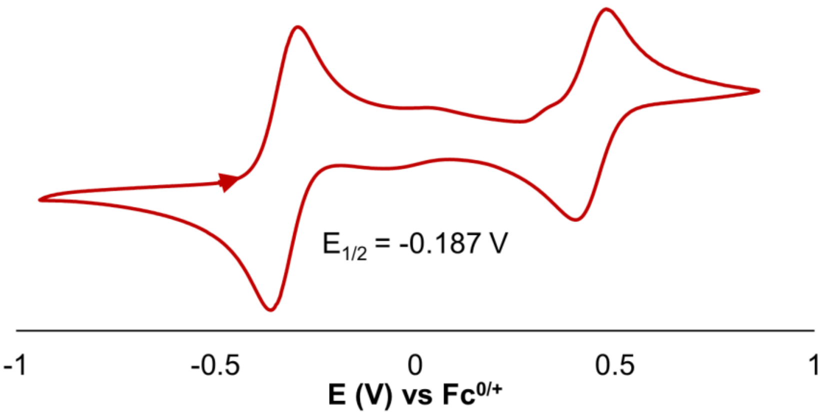

Figure S8. Cyclic Voltammogram of H_2Phen in MeCN with 0.1 M [$^nBu_4N$][PF_6] supporting electrolyte. The small events from -0.25 V to 0.25 V belong to the dehydrogenated product phenazine, which forms upon oxidation of the substrate.

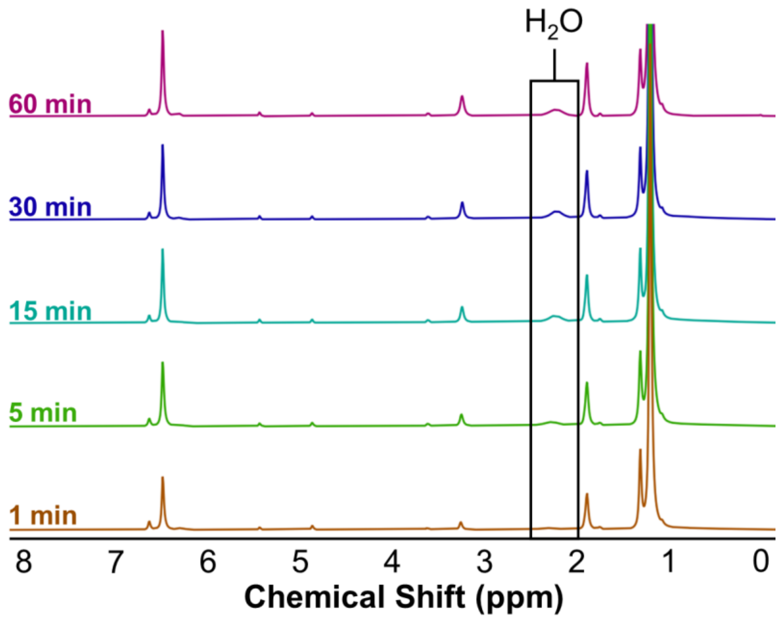

Figure S9. Timepoint analysis of the diamagnetic region of the ^{1}H NMR spectra of $\mathbf{V_6O_7}^{\mathbf{1+}}$ with $^tBu_2$HQ in CD_3CN at 21 °C.

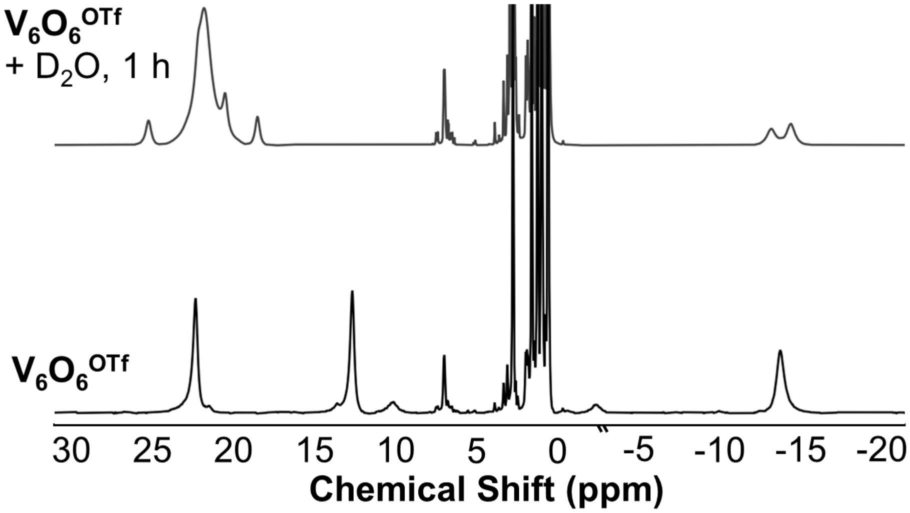

Figure S10. ^{1}H NMR spectrum of $[V_6O_6(OCH_3)_{12}]^{OTf}$ before and after reaction with degassed D_2O in CD_3CN at 21 °C.

Table S3. Bond valence sum calculations for $\mathbf{V_6O_6^{1+}}$ based on X-Ray crystallographic data collected at 100 K. Table reflects the results of BVS calculations using V-E (E = O, N) valence parameters (r_0) for different oxidation states of vanadium.

$\mathbf{V_6O_6^{1+}}$	V1	V2	V3	V4	V5	V6
V(III)	**3.093**	4.516	3.946	4.511	3.949	3.973
V(IV)	3.167	4.623	**4.040**	4.619	**4.043**	**4.067**
V(V)	3.404	**4.920**	4.306	**4.914**	4.310	4.335

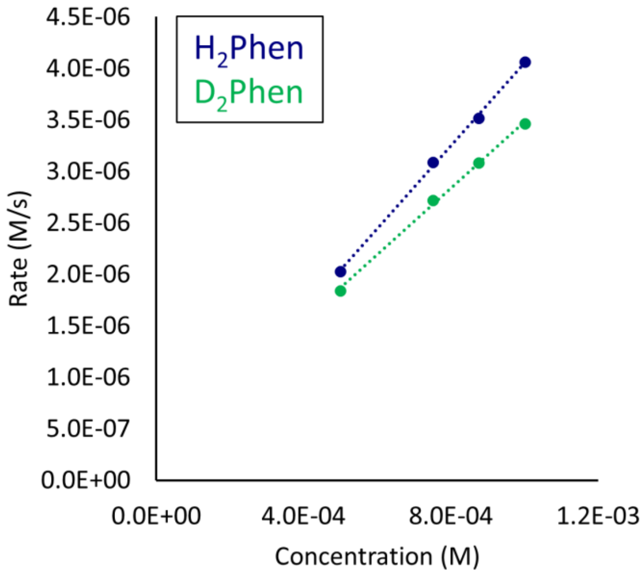

Figure S11. Comparison of the rate of reaction between the neutral cluster, $\mathbf{V_6O_7^0}$, and the reductants H_2Phen (blue) and the deuterated version, D_2Phen (green). Results show a decrease in the rate of reaction upon isotopic substitution, suggesting the hydrogen atom is involved in the rate limiting step. All results were collected by measuring rate of formation of the product, $\mathbf{V_6O_6^0}$, using ^{1}H NMR spectroscopy. All experiments were performed in CD_3CN at 0 °C.

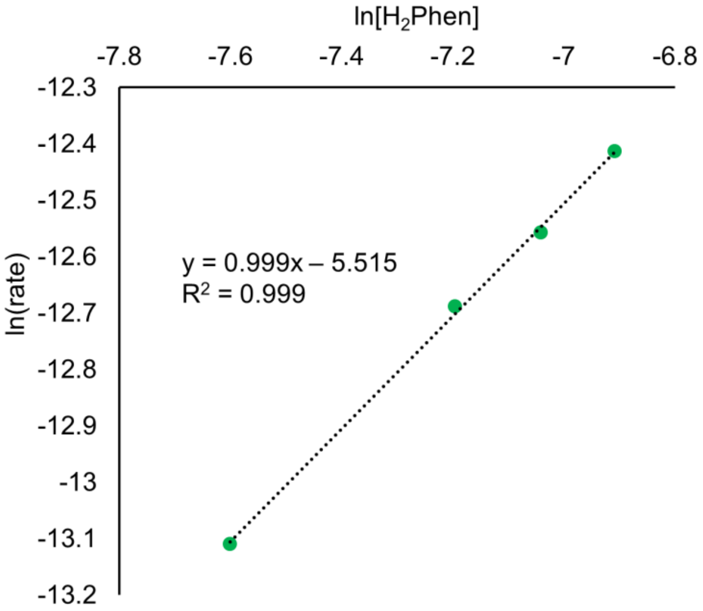

Figure S12. Natural log of the rate plotted against the natural log of the concentration of H_2Phen in the reduction of $\mathbf{V_6O_7^0}$ by H_2Phen. All reactions are performed in MeCN at 0° C. The changes in the rate of formation of $\mathbf{V_6O_6^0}$ are measured as the concentration of H_2Phen is varied from 0.5 – 1 mM. The concentration of $\mathbf{V_6O_7^0}$ is held at 5 mM. The slope of ~1 indicates an order of 1 for the reaction.
